# Extracellular vesicles derived from PPRV-infected cells enhance signaling lymphocyte activation molecular (SLAM) receptor expression and facilitate virus infection

**DOI:** 10.1371/journal.ppat.1010759

**Published:** 2022-09-09

**Authors:** Yan Chen, Ting Wang, Yang Yang, Yuan Fang, Bao Zhao, Wei Zeng, Daiyue Lv, Leyan Zhang, Yanming Zhang, Qinghong Xue, Xiwen Chen, Jingyu Wang, Xuefeng Qi

**Affiliations:** 1 College of Veterinary Medicine, Northwest A&F University, Yangling, Shaanxi, China; 2 Shaanxi Animal Disease Control Center, Xi’an, China; 3 China Institute of Veterinary Drug Control, Beijing, China; 4 Animal Disease Prevention and Control & Healthy Breeding Engineering Technology Research Center, Mianyang Normal University, Mianyang, Sichuan, China; Thomas Jefferson University - Center City Campus: Thomas Jefferson University, UNITED STATES

## Abstract

Peste des petits ruminants virus (PPRV) is an important pathogen that seriously influences the productivity of small ruminants worldwide. PPRV is lymphotropic in nature and SLAM was identified as the primary receptor for PPRV and other *Morbilliviruses*. Many viruses have been demonstrated to engage extracellular vesicles (EVs) to facilitate their replication and pathogenesis. Here, we provide evidence that PPRV infection significantly induced the secretion levels of EVs from goat PBMC, and that PPRV-H protein carried in EVs can enhance SLAM receptor expression in the recipient cells via suppressing miR-218, a negative miRNA directly targeting SLAM gene. Importantly, EVs-mediated increased SLAM expression enhances PPRV infectivity as well as the expression of various cytokines related to SLAM signaling pathway in the recipient cells. Moreover, our data reveal that PPRV associate EVs rapidly entry into the recipient cells mainly through macropinocytosis pathway and cooperated with caveolin- and clathrin-mediated endocytosis. Taken together, our findings identify a new strategy by PPRV to enhance virus infection and escape innate immunity by engaging EVs pathway.

## Author summary

Peste des petitsruminants virus (PPRV) infection induces a transient but severe immunosuppression in the host, which threatens both small livestock and endangered susceptible wildlife populations in many countries. Despite extensive research, the mechanism underlying pathogenesis of PPRV infection remains elusive. Our data provide the first direct evidence that the EVs derived from PPRV-infected cells are involved in PPRV replication. In this study, the EVs derived from PPRV-infected goat PBMCs can enhance SLAM expression in the recipient cells, and more importantly, EVs-mediated increased SLAM expression enhances PPRV replication as well as the expression of various cytokines related to SLAM signaling pathway in the recipient cells. Taken together, our research has provided new insight into understanding the effect of EVs on PPRV replication and pathogenesis, and revealed a potential therapeutic target for antiviral intervention.

## Introduction

Peste des petits ruminants (PPR) is a highly contagious fatal disease in domestic and wild small ruminants [1]. The causative agent of PPR, Peste des petits ruminants virus (PPRV), belongs to the *Morbillivirus* genus [2]. PPRV has six structural proteins, including the nucleocapsid (N), matrix (M), phosphoprotein (P), fusion (F), hemagglutinin (H), and polymerase (L) proteins, and two nonstructural proteins C and V, which perform multiple roles in the pathogenicity of PPRV and counteract host antiviral responses [3]. Both sheep and goat are susceptible to PPRV, while goat is naturally more susceptible to PPRV due to the host- or virus-derived factors [4–6]. Like all morbilliviruses, PPRV has an established lymphatic and epithelial tropism [7,8] and PPRV infection usually caused severe suppression of immune responses in host [9–11].

Signaling lymphocyte activation molecules (known as SLAM or CD150) expressed on the surface of lymphocytes act as primary receptor for morbilliviruses entry, including MV, RPV, CDV, and PPRV [12–14]. Our and other studies have demonstrated that PPRV infection induced transient increased SLAM expression in goat PBMCs during early infection, and its expression levels is closely associated with the levels of PPRV proliferation [15–17]. Importantly, we found that PPRV hemagglutinin protein (H) increased SLAM expression through suppressed miR-218 expression, a negative miRNA directly targeting SLAM gene [16]. It is interesting to note that PPRV infection not only caused a rapid increased SLAM expression in individual infected, but also in neighboring uninfected cells, which imply that PPRV-infected cells may contribute to the regulation of SLAM receptor expression on adjacent cells via intercellular communication [16]. In addition, SLAM signaling has been reported to function as a modifier in immunodeficiency disease [15,18,19]. It has been implied that SLAM signaling may play a key role in mediating the strong immunosuppression induced by the measles virus [20–22].

Extracellular vesicles (EVs) have been characterized as bioactive vesicles that function to promote intercellular communication [23]. Although EVs and their contribution to replication and pathogenesis of viruses remain largely unexplored, a number of RNA viruses have been investigated in the field, including PRRSV, HBV, HCV and Dengue virus [24–27]. The EVs derived from virus infected cells containing altered composition confers numerous novel functionalities [28,29], which can be transferred to the recipient cells and modulate their functions. It is known that endocytosis is the primary method for uptake of EVs by the recipient cells [24,30]. In addition, several studies have suggested that EVs exploit the virus entry machinery and pathway to transmit IFN-α-induced antiviral activity [31,32]. Although many studies highlight the potential and crucial roles of EVs in viral transmission and infection, the possible roles of EVs derived from PPRV-infected cells in the PPRV pathogenesis has not been explored.

In this study, we investigated the effect of EVs derived from PPRV-infected cells on SLAM expression in the recipient cells and identified the mechanism that may play a key role in thepathogenesis of PPRV infection.

## Results

### PPRV infection increases extracellular vesicles secretion

**Fig 1:**
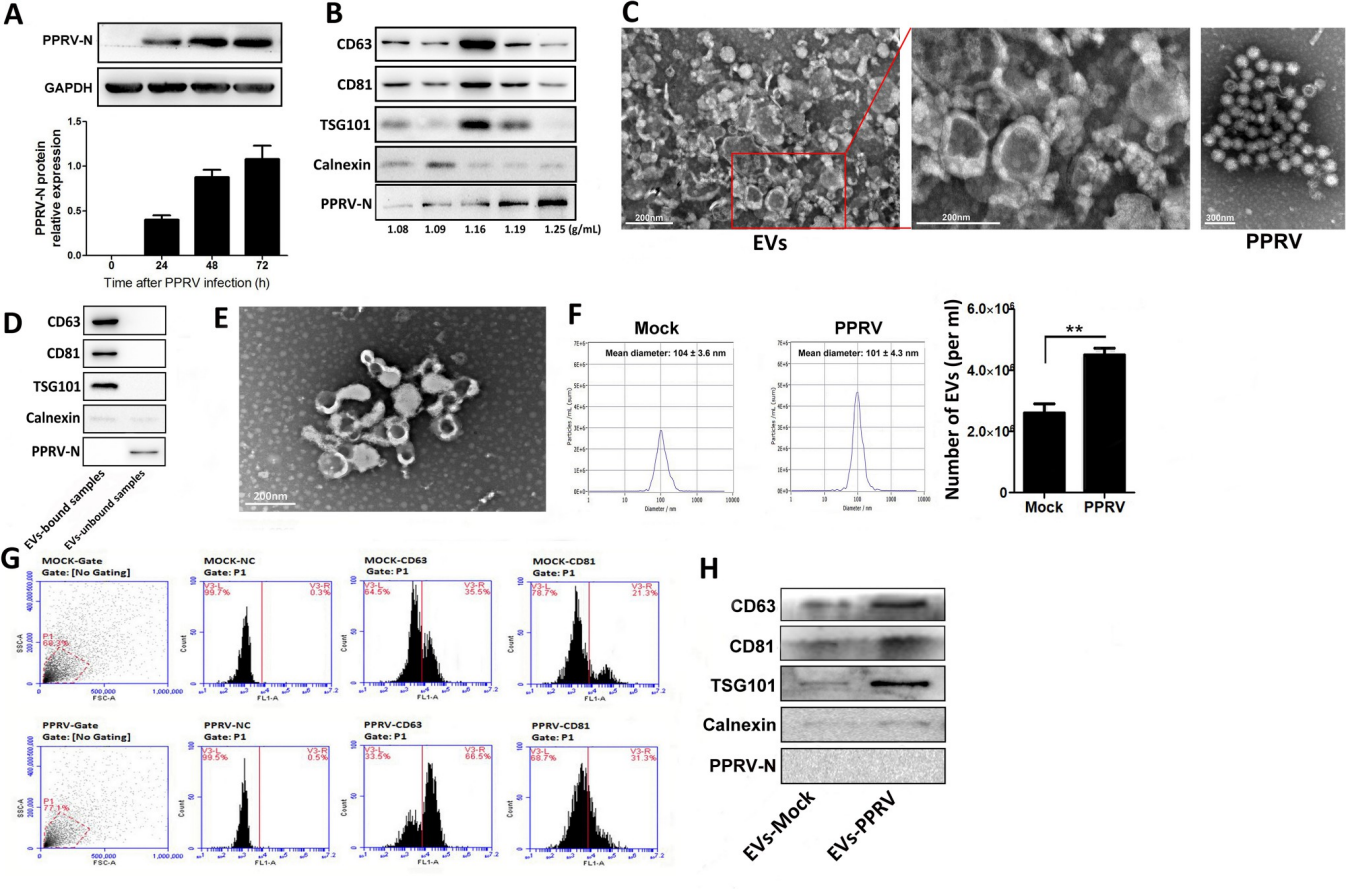
PPRV infection increases extracellular vesicles secretion. (A) Western blot analysis of N protein in PPRV-infected (MOI = 1) and mock-infected goat PBMCs. Equal amounts of protein from PPRV to mock-infected cells were separated using SDS-PAGE and transferred to PVDF membranes. The membranes were probed with N antibody. (B) Extracellular vesicles (EVs) in the supernatants of PPRV-infected (MOI = 1) goat PBMCs at 24 hpi were purified by density gradient separation and analyzed by Western blot for positive EVs marker CD63, CD81, and TSG101, and negative EVs marker Calnexin, as well as PPRV N protein expression. (C) Extracellular vesicles in the supernatants of PPRV-infected (MOI = 1) goat PBMCs at 24 hpi were purified by density gradient separation and analyzed by transmission electron microscopy (TEM). Representative image of purified EVs and free PPRV is shown. (D) Extracellular vesicles purified from PPRV-infected (MOI = 1) goat PBMCs at 24 hpi by density gradient separation in combined with CD63 immunomagnetic bead separation wereanalyzed by Western blot for positive EVs marker CD63, CD81, and TSG101, and negative EVs marker Calnexin, as well as PPRV N protein expression. (E) Extracellular vesicles in the supernatants of PPRV-infected (MOI = 1) goat PBMCs at 24 hpi were purified by density gradient separation in combined with CD63 immunomagnetic bead separation, and were analyzed by TEM. Representative image of purified EVs is shown. (F) Nanoparticle Tracking Analysis (NTA) shows absolute number of purified EVs in per ml cell culture supernatants from mock- and PPRV-infected cells, and size of EVs was 100 nm ± 10 nm. (G) Flow cytometry analysis of EVs positive marker CD63 and CD81. Purified EVs were bound to beads with a size that can be detected by direct sorting and then labeled with fluorophore-conjugated primary antibodies or matched isotype controls and analyzed by flow cytometry. EVs from mock-infected (*top*) and PPRV-infected (*bottom*) cells were positive for both CD63 and CD81. (H) Purified EVs derived from mock- or PPRV-infected cells were analyzed on Western blots probed with antibody direct against EVs positive marker CD63, CD81, and TSG101, and negative EVs marker Calnexin, as well as PPRV N protein. Data are given as means ± standard deviation (SD) from three independent experiments. *P* values were calculated using Student’s t test. An asterisk indicates a comparison with the indicated control. *, *P* < 0.05; **, *P* < 0.01.

Extracellular vesicles have been shown to excrete from cells infected by viruses and deliver various protein and RNA molecules to neighboring cells and modulating host immune responses [26,33,34]. Here, we isolated EVs from the supernatants of the mock- and PPRV-infected goat PBMCs. We selected 24 hpi as the time point for the isolation of EVs from PPRV-infected cells due to the higher viral loads (Fig 1A). To obtain EVs with great purity and exclude contamination with PPRV particles, we first isolated EVs by density gradient centrifugation technique as described previously [35]. Western blot analysis of 1 ml fractions following density gradient separation indicated that PPRV-infected PBMCs derived EVs were enriched at a buoyant density of 1.16 g/ml, based on the strong expression of representative EVs markers CD63, CD81, and TSG101, while weak expression of negative marker for EVs, Calnexin (Fig 1B). This density is consistent with that reported for EVs isolated from a diverse range of cell types [35]. However, the presence of PPRV N protein, in 1.16 g/ml density fraction suggests the incomplete separation of EVs from viruses by density-based separation method. Transmission electron microscopy (TEM) analysis showed that the purified EVs by density gradient separation display a cup-shaped appearance ranging from about 60 to 150 nm in size (Fig 1C), which is consistent with published reports for EVs morphology [35]. Moreover, the purified PPRV particles are enveloped, have a spherical appearance with asize of about 150 nm (Fig 1C).Then, the isolated EVs by density gradient centrifugation technique were further purified by CD63 immunomagnetic bead affinity purification [26]. StrongEVs marker protein expression and the absence of viral proteins expression in bead bound samples suggest that the immuno affinity isolation technique was the superior strategy for preparation of PPRV-infected cells derived EVs (Fig 1D). TEM analysis of EVs by immunoaffinity isolation revealed a homogeneous population of vesicles round in shape and with a size distribution in the range 90–110 nm (Fig 1E).

To further determine the purity and secretion levels of PPRV associated EVs, we performed particle sizing, flow cytometry, and immunoblot assayto analyze purified EVs released from PPRV- and mock-infected cells. Nanoparticle Tracking Analysis (NTA) showed that these EVs are with diameter distribution between 90–110 nm, corresponding to the measured size of EVs by TEM. This analysis also showed that higher number of EVs from PPRV-infected cells was detected in comparison with that from mock-infected cells (Fig 1F). The purified EVs were further characterized by an analysis of EVs markers with flow cytometry (Fig 1G) and Western blot (Fig 1H). It was clearly showed that PPRV infection significantly induced EVs secretion levels as compared with that of mock-infected cells.

### Extracellular vesicles derived from PPRV-infected goat PBMCs contained viral components

**Fig 2:**
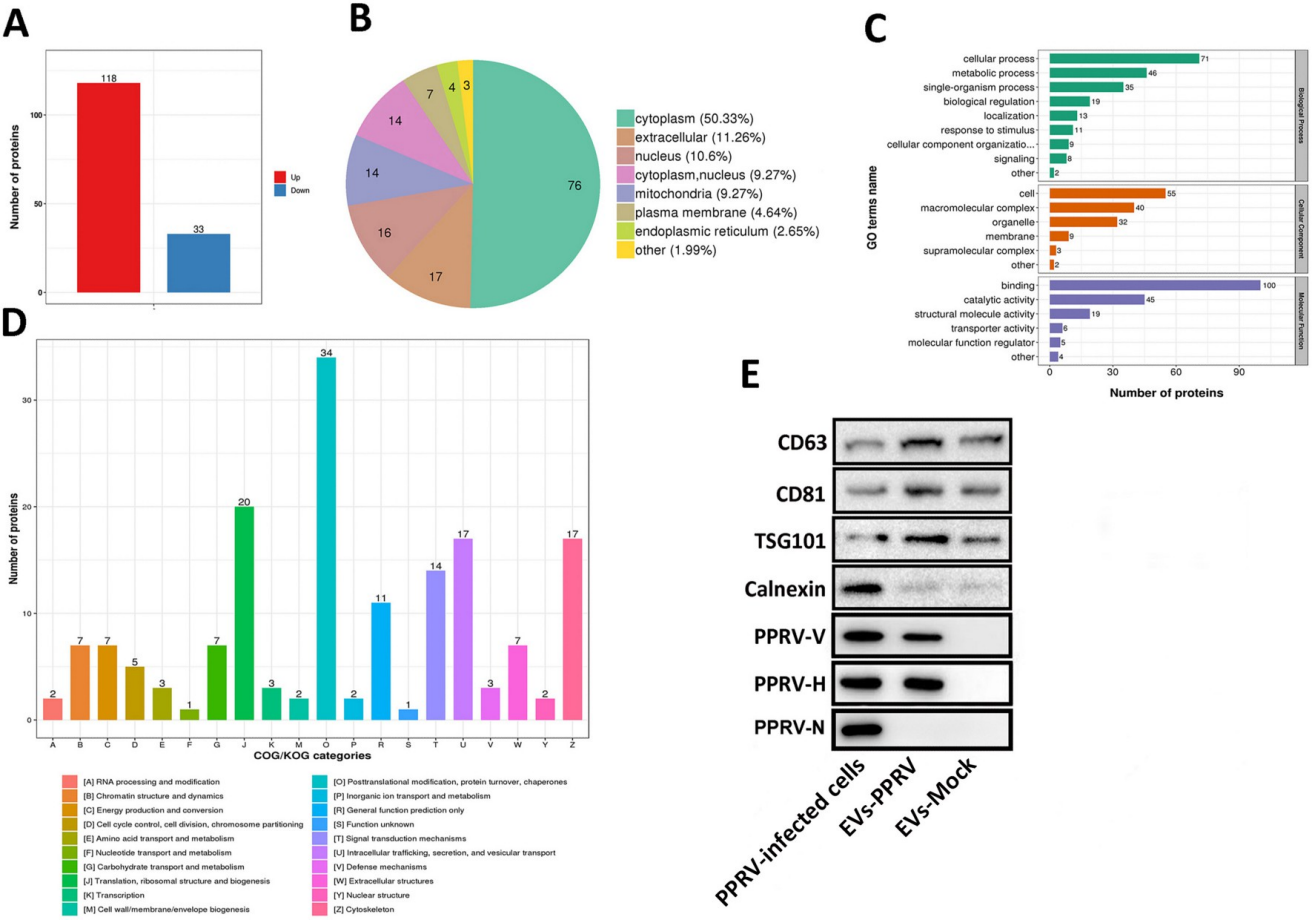
Components of extracellular vesicles derived from PPRV-infected goat PBMCs. (A) Purified EVs derived from PPRV-infected goat PBMCs were analyzed with LC-MS/MS to determine the host proteins and viral proteins present. Collectively, 151 proteins were differentially expressed in EVs derived from PPRV- or mock- infected goat PBMCs. (B) Wolfpsort analysis of subcellular localization of host proteins in PPRV-associated EVs. (C) The GO functional classification of differentially expressed proteins carried by PPRV-associated EVs versus mock-associated EVs were classified into three categories: biological process (9 subcategories), cellular components (6 subcategories), and molecular function (6 subcategories). (D) COG/KOG analysis of functions of differentially expressed protein contained in EVs. Numbers above bars indicate protein amounts. (E) PPRV proteins in EVs were confirmed on Western blots probed with antibody direct against PPRV V, PPRV H or PPRV N. The PPRV infected goat PBMCs were used as positive control.

To characterize the contents of EVs purified from PPRV-infected cells, a liquid chromatography-tandem mass spectrometry (LC-MS/MS) analysis was performed. In total, 986 host proteins were identified within the purified EVs, among which 367 proteins were quantified (the proteins which were measurable in at least one group were include as quantifiable proteins in the analysis). Our data showed that 151 proteins (fold change >2.0) were differentially expressed in isolated EVs derived from PPRV-infected group compared with mock-infected group. Among the differentially expressed proteins, 118 were upregulated and 33 were downregulated (Fig 2A, S1 Table). Subcellular location analysis of differentially expressed proteins contained in EVs revealed that about 50.33% of these proteins were cytoplasm in origin, 11.26% proteins were annotated as belonging to extracellular proteins, whereas the other categories were from nucleus (10.6%), mitochondria (9.27%), both cytoplasm and nucleus (9.27%), plasma membrane (4.64%) and endoplasmic reticulum (2.65%) (Fig 2B). To clarify the function of differentially expressed proteins in EVs, we analyzed the distribution of differentially expressed proteins in Gene Ontology (GO) terms and Kyoto Encyclopedia of Genes and Genomes (KEGG) database. These proteins predominantly participated in 9 biological process categories, 6 molecular function categories and 6 cellular component categories. The annotated proteins were mainly involved in biological processes associated with cellular process, metabolic process, single-organism process, and biological regulation (Fig 2C). To further determine the functional classification of the differentially expressed proteins in EVs, we performed Clusters of Orthologous Groups of proteins (COG/KOG) analysis of proteins. The COG/KOG categorical analysis of differentially expressed proteins included 20 major biological functions, and the top 3 major protein functions were defined as "Posttranslational modification, protein turnover, chaperones", "Translation, ribosomal structure and biogenesis", " Intracellular trafficking, secretion, and vesicular transport", and "Cytoskeleton" (Fig 2D). Previous studies have suggested that viral genomes and/or viral proteins are present in EVs from cells infected by several viruses, including HCV and hepatits B virus [36,37]. LC-MS/MS analysis revealed the presence of N and H protein of PPRV in the purified EVs from PPRV-infected cells. Western blot analysis also showed that the EVs derived from PPRV-infected PBMCs contained PPRV V and H protein (Fig 2E). To further investigate whether PPRV-associated EVs contain viral genomic RNA, real-time quantitative PCR was performed to detect PPRV N genein EVs prepared from PPRV-infected cells. Our data showed that PPRV N gene was not detected in PPRV-associated EVs. Taken together, these results indicate that proteins were differentially expressed in EVs from PPRV-infected PBMCs compared with mock-infected, and the EVs isolated from the supernatants of PPRV-infected PBMCs contain viral proteins.

### PPRV associate extracellular vesicles enhance SLAM expression in the recipient cells

**Fig 3:**
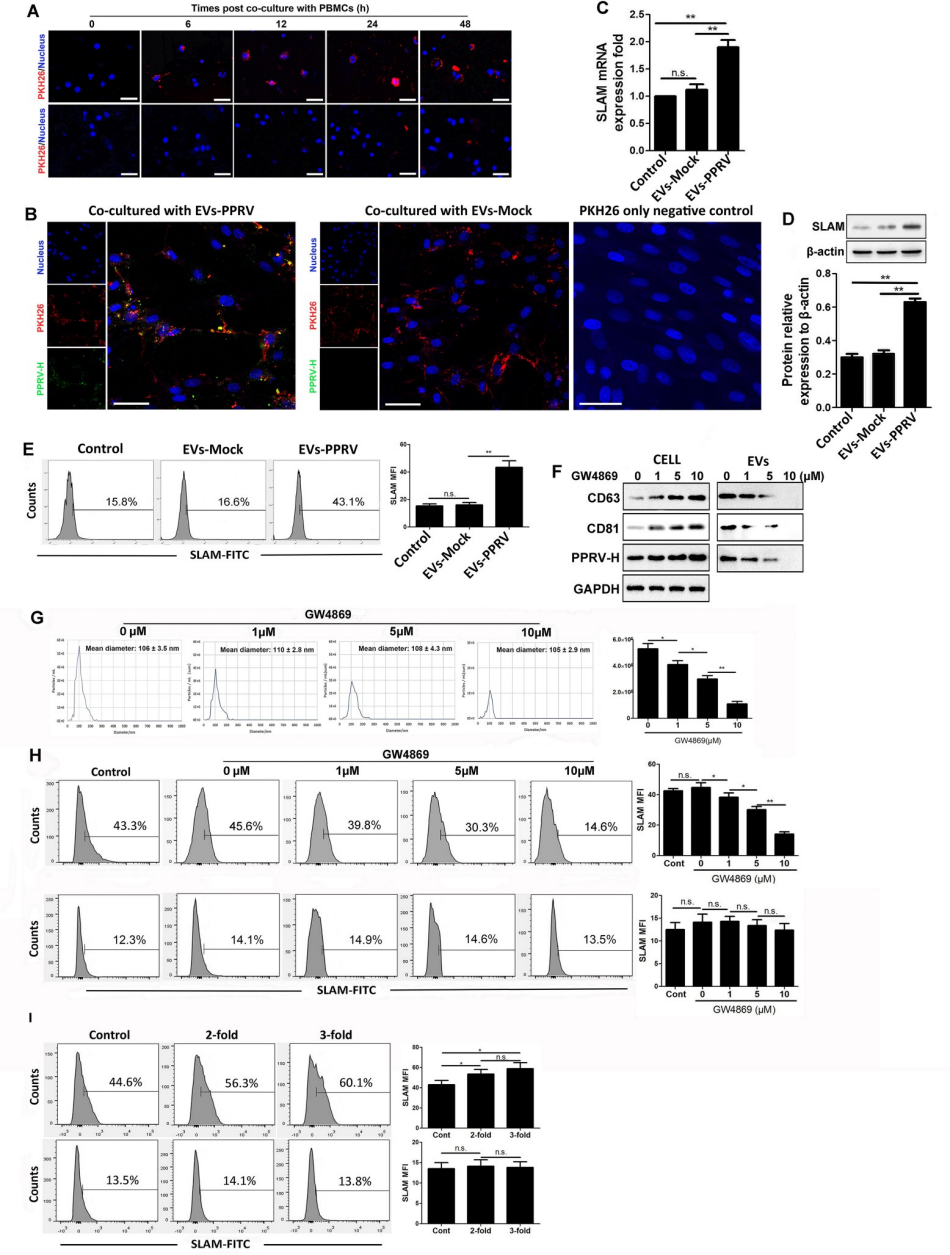
PPRV associate extracellular vesicles enhance SLAM expression in the recipient cells. (A) PKH26-labeled EVs internalization by goat PBMCs (*top*); PKH26-labeled PBS were used as PKH26-only negative control (*bottom*) (Scale bar = 30 μm). (B) EVs isolated from PPRV-infected cells (EVs-PPRV) or from Mock-infected cells (EVs-Mock)were labeled with PKH26, and co-cultured with naive goat PBMCs for 48 h, immunofluorescent staining was performed to analysis the internalization of EVs and PPRV H protein expression in the recipient cells. PKH26-labeled PBS were used as PKH26-only negative control (Scale bar = 30 μm). (C, D, E) Equal quantities of EVs-PPRV or EVs-Mock were respectively co-cultured with the same amount of naive goat PBMCs for 48 h, and SLAM expression levels in the recipient cells were determined by RT-PCR (C), Western blot (D), and flow cytometry (E). Untreated goat PBMCs were used as the blank control. (F, G) Goat PBMCs were infected with PPRV at an MOI of 1 for 1 h and then maintained in medium containing indicated concentrations of GW4869 for 48 h. Then, the cells and the EVs isolated from the supernatants were subjected to Western blot for CD63, CD81, and PPRV H protein expression (F), and EVs were also subjected to NTA analysis(G). (H) Equal quantities of goat PBMCs infected with PPRV (MOI = 1) or Mock infected for 1 h and then maintained in medium containing indicated concentrations of GW4869 for 48 h. Then, the EVs from PPRV-infected cells (*top*) or Mock-infected cells (*bottom*) were respectively incubated with naive goat PBMCs that have the same amount of EVs-producing cells for 48 h and subjected to flow cytometry for SLAM expression. (I) Different fold number of EVs-PPRV were incubated with naive goat PBMCs for 48 h, and cells were harvested and subjected to flow cytometry for SLAM expression (*top*). Equal quantities of EVs-Mock incubated with the same amount of PBMCs were used as control (*bottom*). GAPDH was used as a loading control in RT-PCR and Western blot analysis. Data are given as means ± standard deviation (SD) from three independent experiments. *P* values were calculated using Student’s *t* test. An asterisk indicates a comparison with the indicated control. *, *P*<0.05; **, *P*<0.01; n.s., not significant.

It has previously been showed that PPRV H protein can regulate miR-218-mediated SLAM expression [16]. The high load of PPRV H protein carried by EVs derived from PPRV-infected goat PBMCs (EVs-PPRV) prompted us to investigate whether EVs-PPRV can transmit viral proteins to the recipient cells and regulate response of cells. To this end, we first assessed whether EVs-PPRV is indeed internalized in naive goat PBMCs. Purified EVs-PPRV were labeled with the fluorescent lipid dye PKH26 and incubated with naive goat PBMCs and examined for EVs uptake by indirect immunofluorescence assay (IFA). Our data showed that EVs-PPRVwere internalized in the recipient cellsin a co-culture time-dependent manner (Fig 3A, *top*). No positive fluorescence was detected in cells incubated with PKH26-labeled phosphate buffered saline (PBS) (PKH26-only negativecontrol)confirmed that the signal is specific to labeled EVs (Fig 3A, *bottom*). Importantly, IFA analysis confirmed the presence of H protein in the recipient cells co-culture with EVs-PPRV (Fig 3B), which suggesting EVs-mediated transfer of PPRV H protein. To determine the effect of internalized EVs-PPRV on modulation of SLAM expression in the recipient cells, naive PBMCs were co-cultured with EVs-PPRV for 48 h and SLAM mRNA expression was analyzed by reverse transcription (RT)-PCR. A significant increase of SLAM mRNA expression in cells cu-cultured with EVs-PPRV was detectedcompared tothat incubated with EVs from mock-infected cells (EVs-Mock) or untreated control cells (Fig 3C). Similar results were obtained by Western blot (Fig 3D) and flow cytometry (Fig 3E).

To further investigate the involvement of the EVs pathway in the enhancement of SLAM expression in the recipient cells, we examined the effects of an inhibitor of EVs release, GW4869, on EVs-mediated SLAM expression in the recipient cells. MTT assay demonstrated no obvious cytotoxicity at indicated concentrations of GW4869 tested in goat PBMCs. Our data showed that, as the concentration of GW4869 increased, the amount of released EVs and PPRV H protein carried by EVs gradually declined (Fig 3F). NTA analysis also showed that the number of EVs from PPRV-infected cells treated with different concentrations of GW4869 decreased in a GW4869 dose-dependent manner (Fig 3G). Correspondingly, the SLAM expression levels on the recipient cells incubated with EVs-PPRV from the cells treated with different concentrations of GW4869 decreased in a GW4869 dose-dependent manner (Fig 3H). However, GW4869 has no effect onSLAM expression in the recipient cells incubated with EVs-Mock (Fig 3H). To exclude the effect of EV numbers on SLAM expression in the recipient cells, equal quantities of EVs-PPRV or EVs-Mock were co-cultured with the same amount of naive goat PBMCs and SLAM expression in the recipient cells was detected by flow cytometry. Our data showed that treatment of cells with more fold number of EVs-PPRV increased SLAM expression (Fig 3I, *top*), while treated with more fold number of EVs-Mock has no significant effects on SLAM expression (Fig 3I, *bottom*). Taken together, these results suggest that EVs-PPRV can transmit PPRV H protein to naive goat PBMCs. Importantly, EVs-PPRV can upregulate SLAM expression in the recipient cells.

### PPRV H protein contained in extracellular vesicles is sufficient to regulate miR-218-mediated SLAM expression in the recipient cells

**Fig 4:**
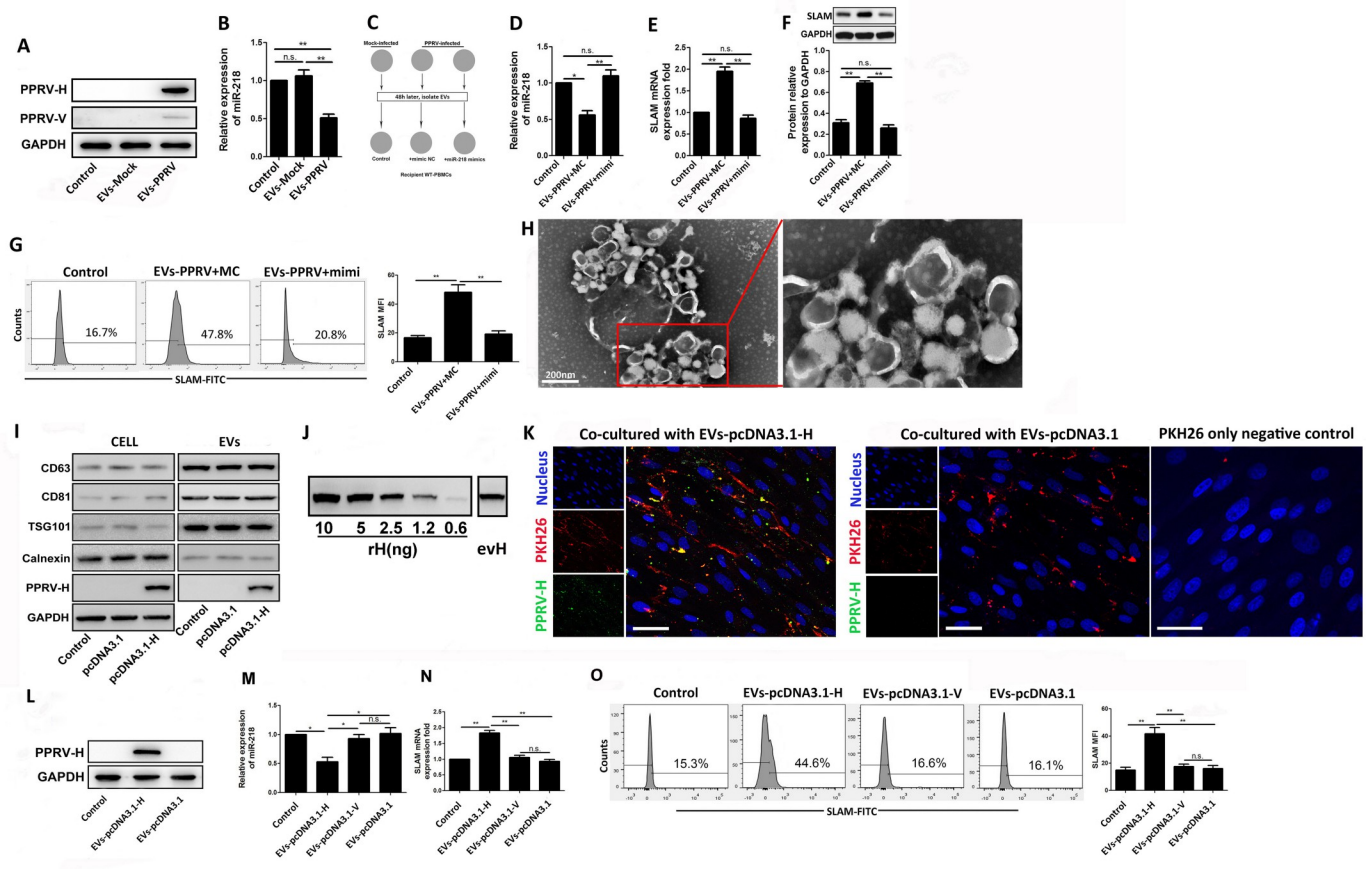
PPRV-H protein contained in extracellular vesicles is sufficient to regulate miR-218-mediated SLAM expression in the recipient cells. (A, B) Extracellular vesicles isolated from the supernatants of PPRV-infected (EVs-PPRV) and mock-infected goat PBMCs (EVs-Mock) were incubated with naive goat PBMCs, after 48 h, viral proteins and miR-218 expression levels in the recipient cells were detected by Western blot (A) and qRT-PCR (B), respectively. (C) Schematic presentation of the effects of PPRV-associated EVs on miR-218-mediated SLAM expression experiment. (D, E, F, G) Equal quantities of EVs-PPRV or EVs-Mock were co-cultured with the same amount of naive goat PBMCs pretransfected with either miR-218 mimic or control mimic (MC) for 48h. Untreated group of cells were used as control. Then, the expression of miR-218 (D) and SLAM mRNA (E) in the cells were analyzed by RT-qPCR, and SLAM expression was further detected by Western blot (F) and flow cytometry (G). (H) Transmission electron microscopy (TEM) analysis of EVs from goat PBMCs transfected with plasmids expressing PPRV H protein for 48 h.(I) Goat PBMCs were transfected with plasmids expressing PPRV H protein for 48 h, and then subjected to Western blot for the analysis of the expression of positive EVs marker CD63, CD81 and TSG101, and negative EVs marker Calnexin, as well as PPRV H in the cells and the isolated EVs, respectively. (J) Western blot for the indicated amounts of recombinant PPRV H (rH) and in a typical preparation of PPRV H-containing EVs (evH) (10 μg of EVs protein). (K) EVs from goat PBMCs transfected with plasmids expressing PPRV H protein (EVs-pcDNA3.1-H) were labeled with PKH26 and incubated with naive goat PBMCs for 48 h, immunofluorescent staining was performed to analysis the expression of PPRV H protein (Scale bar = 30 μm). (L, M, N, O) Goat PBMCs were incubated with EVs-pcDNA3.1-H, EVs-pcDNA3.1,or EVs from pcDNA3.1-V transfected cells (EVs-pcDNA3.1-V) for 48 h, and then subjected to Western blot for the analysis of the expression PPRV H protein (L), and the expression of miR-218 (M) and SLAM mRNA (N) were detected by RT-qPCR, and flow cytometry was performed to analyze SLAM expression on the surface of the cells (O). GAPDH was used as a loading control in RT-PCR and Western blot analysis. Data are given as means ± standard deviation (SD) from three independent experiments. *P* values were calculated using Student’s *t* test. An asterisk indicates a comparison with the indicated control. *, *P*<0.05; **, *P*<0.01; n.s., not significant.

Our previous studies have documented that PPRV H protein alone can increase SLAM receptor expression through down regulation of miR-218 expression in goat PBMCs [16]. To gain insight into the role of PPRV H protein carried by PPRV associated EVs in enhancing SLAM expression on the recipient cells, we first incubated naive PBMCs with EVs-PPRV or EVs-Mock for 48 h and analyzed the expression of PPRV H protein and miR-218 in the recipient cells. A high expression of PPRV H protein and a mild PPRV V protein expression were observed in cells incubated with EVs-PPRV, while no bands were detected in cells incubated with EVs-Mock or in untreated control cells (Fig 4A). Conversely, a significantly decreased miR-218 expression was detected in cells incubated with EVs-PPRV compared to cells incubated with EVs-Mock or untreated control cells (Fig 4B). To further determine whether the increased SLAM expression by EVs-PPRV is miR-218-dependent, EVs-PPRV were incubated with cells pretransfected with miR-218 mimic or mimic control, and EVs-Mock incubated with untreated cells were used as control, as outlined in Fig 4C. Our data showed that a significant decreasedmiR-218 expression levels was detected in EVs-PPRV treated cells pretransfected with control miRNA (MC) compared with control cells, while pretransfection with miR-218 mimic (mimi) reverse the decreased miR-218 expression (Fig 4D). Conversely, a significant increased SLAM mRNA expression was observed in EVs-PPRV treated cells pretransfected with MC compared to miR-218 mimic transfected or control cells (Fig 4E). Similar results were detected by Western blot (Fig 4F) and flow cytometry (Fig 4G).

In order to investigate whether PPRV H protein contained in EVs-PPRV could cause miR-218-mediated up regulation of SLAM in the recipient cells, we transfected goat PBMCs with pcDNA3.1-H or control plasmid for 48 h, and isolated EVs from transfected and untransfected control cells. TEM analysis of prepared EVs revealed a homogeneous population of vesicles round in shape and with a size distribution in the range 90–110 nm (Fig 4H). A significant PPRV H protein expression in EVs from pcDNA3.1-H transfected cells (EVs-pcDNA3.1-H) confirmed PPRV H protein can be packaged into EVs (Fig 4I). There was no detectable PPRV H protein expression in EVs from control plasmid transfected cells (EVs-pcDNA3.1) or untreated control cells (Fig 4I). Furthermore, the EVspositive markers, including CD63, CD81, and TSG101, were enriched, while negative marker Calnexin were weak in the isolated EVs compared with its abundance in the cells (Fig 4I). To measure the concentration of H protein in PPRV H-containing EVs (evH), we generated a calibration curve using purified non-myristoylated recombinant H (rH) produced using bacterial expression system, and quantitated the density of bands corresponding to recombinant and EVs H using Western blot (Fig 4J). The PPRV H content of the EVs showed considerable variability from one preparation to another, however, on average there was 0.5 ng H per 1 μg of total EVs protein. In the experiments reported here, PPRV H concentration was determined for each individual preparation of EVs.

To investigate whether EVs-pcDNA3.1-H can transmit PPRV H protein to recipient cells, we labeled EVs-pcDNA3.1-H with PKH26, and incubated them with naive goat PBMCs. IFA analysis showed that EVs-pcDNA3.1-H and PPRV H protein were co-localized in the cytoplasm of cells after 48 h of incubation (Fig 4K). Western blots probed with monoclonal antibody direct against H protein confirmed the presence of PPRV H protein in the cells incubated with EVs-pcDNA3.1-H (Fig 4L). The expression of miR-218 and SLAM mRNA in cells incubated with equal quantities EVs-pcDNA3.1-H or EVs-pcDNA3.1 were quantified with real-time RT-PCR. Untreated cells and cells incubated with EVs from pcDNA3.1-V transfected cells (EVs-pcDNA3.1-V) were used as control. Our data showed that a significant decreased miR-218 expression was detected in EVs-pcDNA3.1-H incubated cells compared to EVs-pcDNA3.1, or EVs-pcDNA3.1-Vtreated cells and untreated control cells (Fig 4M). Conversely, a significant increased SLAM mRNA expression was detected in cells incubated with EVs-pcDNA3.1-H as compared with cells incubated with EVs-pcDNA3.1, EVs-pcDNA3.1-V, or untreated control cells (Fig 4N). Flow cytometry analysis of the surface expression of SLAM was consistent with the results of qRT-PCR (Fig 4O). Taken together, these results suggest that PPRV H protein contained in EVs is sufficient to regulate miR-218-mediated SLAM expression in the recipient cells.

### PPRV associate EVs promote PPRV replication in the recipient cells

**Fig 5:**
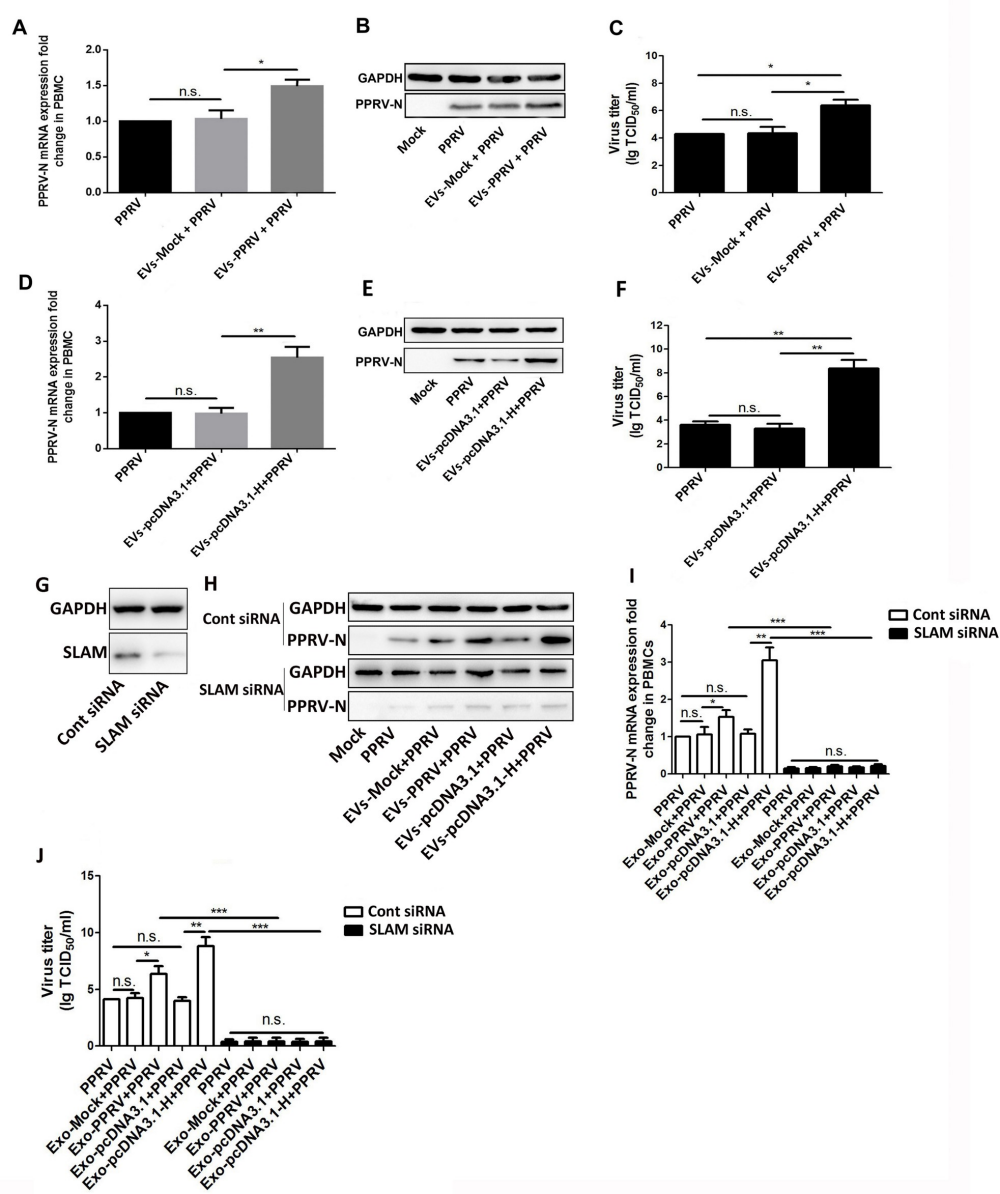
PPRV associate extracellular vesicles promote PPRV replication in the recipient cells. (A, B, C) Extracellular vesicles were purified from PPRV-infected (EVs-PPRV) or Mock-infected (EVs-Mock) goat PBMCs at 24 hpi and co-cultured with naive goat PBMCs for 48h. Then, the cells were infected with PPRV at an MOI of 1 for 24 h, and qRT-PCR (A), Western blot (B), and TCID_50_ (C) assays were performed to determine the viral replication and progeny. (D, E, F) Extracellular vesicles were isolated from pcDNA3.1-H (EVs-pcDNA3.1-H) or pcDNA3.1 control plasmid (EVs-pcDNA3.1) transfected goat PBMCs at 24 h post transfection and co-cultured with naive goat PBMCs for 48h. Then, the cells were infected with PPRV at an MOI of 1. After 24h infection of PPRV, qRT-PCR (D), Western blot (E), and TCID_50_ (F) assays were performed to determine the viral replication and progeny. (G) Western blotanalysis of SLAM expression in goat PBMCs transfected with siRNA against SLAM or scrambled siRNA for 48 h. (H, I, J) Goat PBMCs were transfected with siRNA against SLAM or scrambled siRNA for 48 h; Then, the cells were co-cultured with EVs-PPRV, EVs-pcDNA3.1-H, or respective control for 48h, and then infected with PPRV at an MOI of 1. After 24 h infection of PPRV, Western blot (H), qRT-PCR (I), and TCID_50_ (J) assays were performed to determine the viral replication and progeny. GAPDH was used as a loading control in qRT-PCR and Western blot analysis. Data are given as means ± standard deviation (SD) from three independent experiments. *P* values were calculated using Student’s t test. An asterisk indicates a comparison with the indicated control. *, *P*<0.05; **, *P*<0.01; ***, *P*<0.001; n.s., not significant.

It has previously been demonstrated that the expression levels of SLAM have closely correlated with the replication levels of PPRV [16,38,39]. To investigate whether PPRV associate EVs affect PPRV replication through enhance SLAM expression, EVs isolated from PPRV infected goat PBMCs (EVs-PPRV) or from mock-infected cells supernatants (EVs-Mock) were incubated with naive PBMCs and 48h later the cells were infected with PPRV at an MOI of 1. After 24h infection of PPRV, the cells were collected and subjected to Western blot, qRT-PCR and TCID_50_ analysis to determine the viral propagation. Goat PBMCs infected with PPRV were used as the positive control. Our data showed that EVs-PPRV significantly enhanced virus replication (Fig 5A and 5B) and progeny (Fig 5C) compared to those of the EVs-Mock incubated or positive control cells. Furthermore, we tested the effect of EVs from pcDNA3.1-H transfected cells (EVs-pcDNA3.1-H) on PPRV levels in the recipient cells. Our data showed that treatment of goat PBMCs with EVs-pcDNA3.1-H significantly enhanced PPRV replication (Fig 5D and 5E) and virus titers (Fig 5F) compared to those of the control cells. To further verify that SLAM expression is associated with the enhancement of PPRV replication by PPRV H protein contained in EVs, cells were transfected with small interfering RNA (siRNA) targeting SLAM followed by co-cultured with EVs-PPRV, EVs-pcDNA3.1-H, or respective control, and then infected with PPRV (MOI = 1). Free PPRV-infected cells were used as the positive control. Western blot analysis showed that SLAM protein expression was effectively inhibited by transfection of siRNA (Fig 5G). Although treatment of control siRNA transfected cells with EVs-PPRVor EVs-pcDNA3.1-H significantly enhanced viral replication (Fig 5H and 5I) and progeny (Fig 5J), knockdown of SLAM expression in the recipient cells significantly abolish the enhanced PPRV replication by PPRV H protein contained in EVs (Fig 5H, 5I and 5J). Altogether, these results clearly showed that PPRV-associated EVs enhanced PPRV replication through enhance SLAM expressionin the recipient cells.

### PPRV associate EVsregulate SLAM mediated cytokines expression in the recipient cells

**Fig 6:**
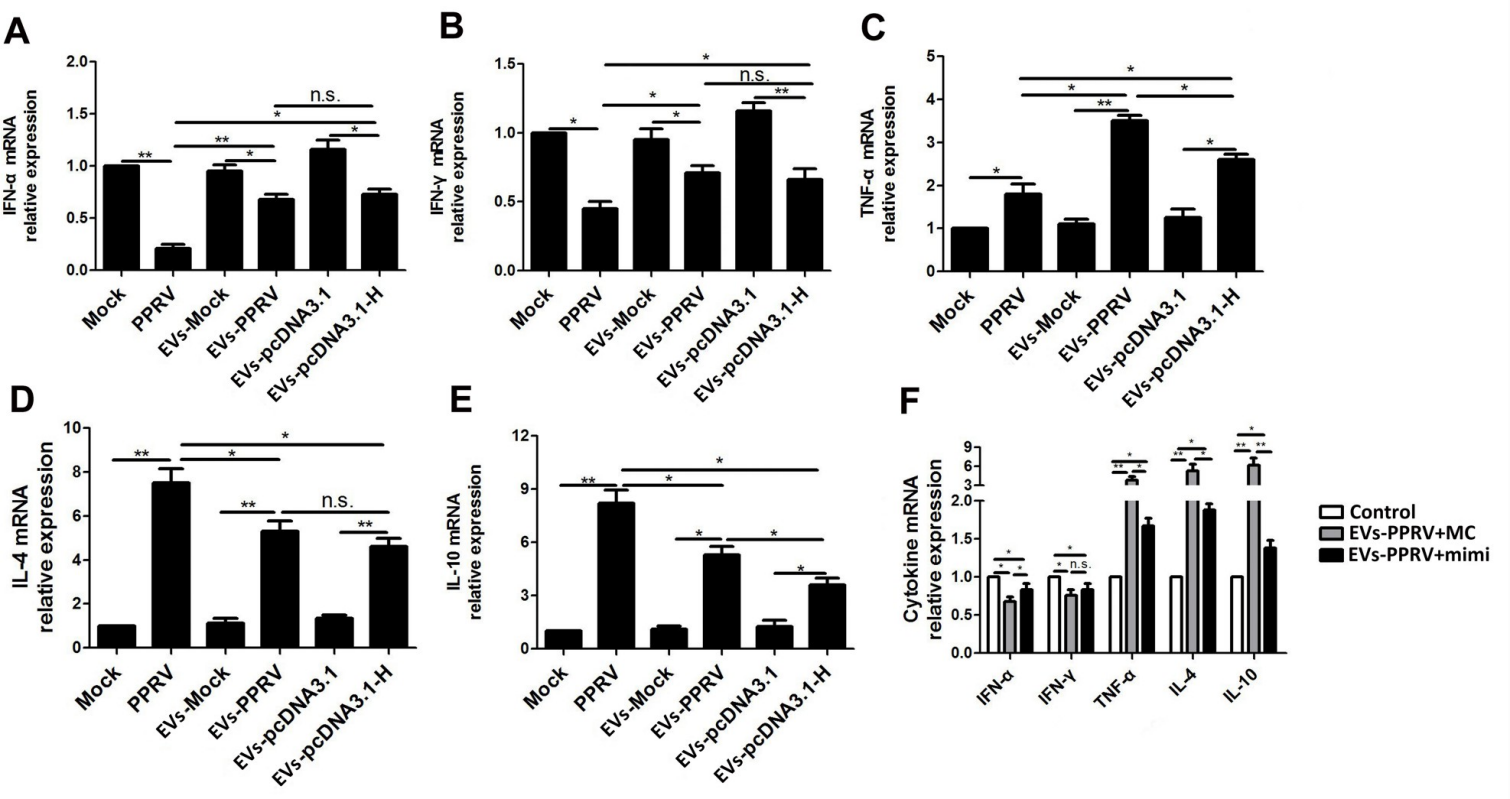
PPRV associate extracellular vesicles regulate SLAM mediated cytokines expression in the recipient cells. (A, B, C, D, E) Goat PBMCs were incubated with extracellular vesicles isolated from mock- (EVs-Mock), PPRV-infected cells (EVs-PPRV), or from cells transfected with pcDNA3.1-H (EVs-pcDNA3.1-H) or pcDNA3.1 control plasmid (EVs-pcDNA3.1). After 48 h, the cells were subjected to qRT-PCR for the analysis of the expression of IFN-α (A), IFN-γ (B), TNF-α (C), IL-4 (D), and IL-10 (E). PPRV-infected cells and Mock-infected cells were used as positive and negative control, respectively. (F) Goat PBMCs were transfected with miR-218 mimic (mimi) or mimic control (MC) for 24 h, and incubated with EVs-PPRV for 24 h. EVs-Mock incubated with untreated cells were used as control. Then, the cells were subjected to qRT-PCR for the analysis of the indicated cytokines expression. GAPDH was used as a loading control in qRT-PCR analysis. Data are given as means ± standard deviation (SD) from three independent experiments. *P* values were calculated using Student’s t test. An asterisk indicates a comparison with the indicated control. *, *P*<0.05; **, *P*<0.01; n.s., not significant.

Because PPRV-induced immunosuppression may attribute to the modulation of SLAM expression and SLAM signaling in lymphocytes [16,39–41], the effect of EVs-PPRV or EVs-pcDNA3.1-H on theexpression of SLAM mediated cytokines bythe recipient cells was determined. The cells were first co-cultured with EVs-PPRV, EVs-pcDNA3.1-H or respective controls. At 48 h post transfection, the change of mRNA expression for indicated cytokines was determined by real-time PCR assays. Mock- and PPRV-infected cells were used as negative and positive control, respectively. Our data showed that PPRV infection significantly suppressed alpha interferon (IFN-α) (Fig 6A) and gamma interferon (IFN-γ) (Fig 6B) expression, while stimulated expressionof tumor necrosis factor α (TNF-α) (Fig 6C), interleukin 4 (IL-4) (Fig 6D), and interleukin 10 (IL-10) (Fig 6E) as previously described [11,16,42–44]. Similar effects of EVs-PPRV or EVs-pcDNA3.1-H on the expression of these cytokines by the recipient cells compared to that of respective control was detected (Fig 6A–6E).

To further determine whether the changed expression of examined cytokines by EVs-PPRV is SLAM mediated, EVs-PPRV were incubated with cells pretransfected with miR-218 mimic (mimi) or mimic control (MC), and EVs-Mock incubated with untreated cells were used as control. Our data showed that similar changes of detected cytokines expression were detected in EVs-PPRV treated cells pretransfected with mimic control as compared with control cells, while pretransfection with miR-218 mimic significantly weakened these effects (Fig 6F). Together, these results demonstrate that PPRV associated EVs can regulate proinflammatory and antiinflammatory cytokines expression in the recipient cells, at least partly, through SLAM-mediated pathway.

### Extracellular vesicles were internalized into the recipient cells via classical endocytic pathway

**Fig 7:**
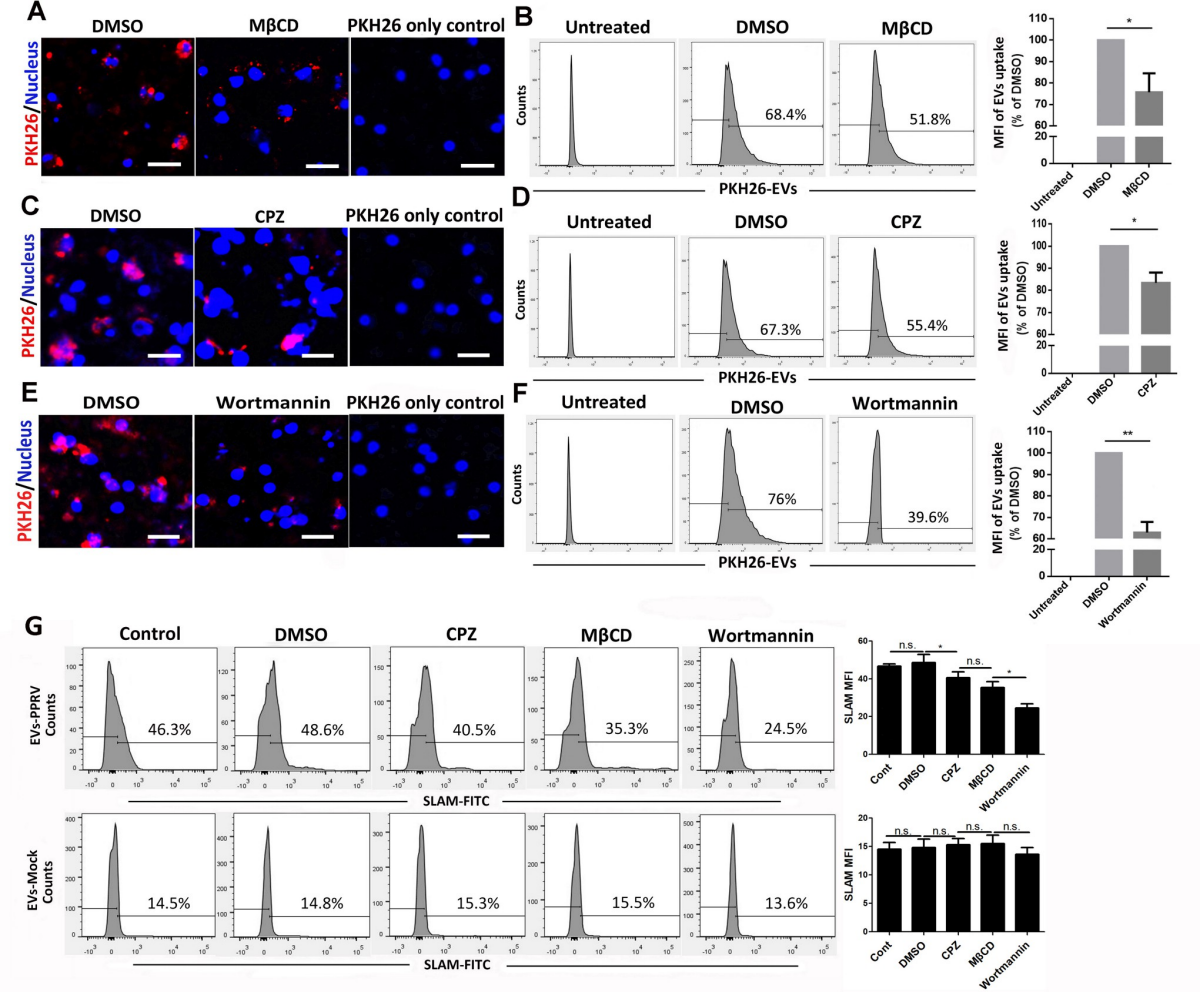
Extracellular vesicles were internalized into the recipient cells via classical endocytic pathway. (A, C, E) Goat PBMCs were pretreated with MβCD (A), CPZ (C), or wortmannin (E) for 1h at 37°C and then incubated with PKH26 labeled-EVs-PPRV for 6h. After removal of surface-bound EVs and ligands, internalized EVs were analyzed by using a confocal laser scanning microscope. The nuclei were stained with Hoechst 33342. PKH26-labeled PBS were used as PKH26-only negative control. (B, D, F) Goat PBMCs were pretreated with MβCD (B), CPZ (D), or wortmannin (F) for 1h at 37°C and then incubated with PKH26 labeled-EVs-PPRV for 6h. After removal of surface-bound EVs and ligands, internalized EVs were analyzed by using flow cytometry. DMSO-treated cells and untreated cells were used as positive and blank control, respectively. (G) Goat PBMCs pretreated with MβCD, CPZ, or wortmannin for 1h were co-cultured with equal quantities of EVs-PPRV or EVs-Mock for 48 h, respectively. Then, SLAM expression on the recipient cells were analyzed by flow cytometry. Scale bar = 30 μm. Data are given as means ± standard deviation (SD) from three independent experiments. *P* values were calculated using Student’s *t* test. An asterisk indicates a comparison with the indicated control. *, *P*<0.05; **, *P*<0.01; n.s., not significant.

Given the emerging roles of PPRV-associated EVs in the enhancement of SLAM expression and regulation of SLAM-mediated cytokines expression in the recipient cells, it is important to understand the transmission mechanisms by which EVs-PPRV are taken up into the recipient cells. It is known that EVs can induce the cellular response of internalization through endocytic pathways [24,30]. Endocytosis occurs via several pinocytic mechanisms that include the caveola-mediated endocytosis (CDE), the clathrin-mediated endocytosis (CME), macropinocytosis, and other, less well-defined mechanisms [45,46]. Membrane cholesterol is required for the formation of caveolae, and is an essential component of lipid rafts. Depletion of cholesterol from the membrane with methyl-*β*-cyclodextran (M*β*CD) can significantly block caveolae-mediated endocytosis [47,48]. To determine whether cholesterol is necessary for EVs entry into goat PBMCs, we use M*β*CD to extract cholesterol from the plasma membrane of cells [49,50]. M*β*CD was used at 5 mM based on the cell viability assay data. Treatment of cells with M*β*CD significantly inhibited PKH26-labeled EVs entry (Fig 7A). Similar results were obtained with Flow cytometry analysis (Fig 7B). These data indicated that EVs entry into goat PBMCs dependent on caveolae and required the involvement of cholesterol.

The clathrin-mediated endocytosis is the uptake of material into cells from the surface using clathrin-coated vesicles. To investigate the role of clathrin in PPRV-associated EVs entry into the recipient cells, CPZ, an inhibitor of clathrin-coated pit assembly, was used to specifically block this pathway [51]. CPZ was used at 10 μM based on the cell viability assay. Both confocal and flow cytometry analysis demonstrated that PKH26-labeled EVs uptake significantly decreased in the presence of CPZ (Fig 7C and 7D), confirming clathrin-dependent endocytosis by EVs internalization.

More than one endocytic route was reported to be used in virus or EVs entry [32,52]. Given the incomplete inhibition of EVs entry by blockade of CDE and CME, there might be alternative pathways to support EVs entry into the recipient cells. It was reported that PI3K is involved in multiple stages of micropinocytosis [51]. To further investigate the role of macropinocytosis in EVs uptake, PKH26-labeled EVs was incubated with goat PBMCs pretreated with DMSO or Wortmannin. Wortmannin treatment at 2.5 μM did not affect cell viability. Confocal images showed that wortmannin treatment obviously inhibitPKH26-labeled EVs entry into cells compared to control cells (Fig 7E), and was confirmed by flow cytometry analysis (Fig 7F).

To determine the role of EVs internalizationin EVs-mediated modulation of SLAM expression in the recipient cells, we pretreated cells with M*β*CD, CPZ, or wortmannin followed by co-cultured with equal quantities of EVs-PPRV or EVs-Mock for 48 h, respectively, and then analyzed SLAM expression by flow cytometry. Our data showed that there is a close correlation between EVs internalization and SLAM expression levels (Fig 7G). Taken together, our data showed that macropinocytosis serve as mainly route for EVs internalization and cooperated with caveolin- and clathrin-mediated endocytosis to ensure EVs-mediated modulation of SLAM expressionin the recipient cells.

## Discussion

Extensive studies have demonstrated that extracellular vesicles play crucial roles in cell communication and in the transfer of genetic information between cells. Although there has been an increasing number of studies on the involvement of EVs in viral pathogenesis and immune responses [27,32,33,53,54], the role of EVs in the infection of *Morbillivirus* genus has largely been unexplored. In the current study, we reveal for the first time that, PPRV infection significantly induced the secretion levels of goat PBMC EVs, and that PPRV H protein carried in EVs enhances SLAM receptor expression in the recipient cells via suppressing miR-218, a negative miRNA directly targeting SLAM gene. Moreover, our data reveal that PPRV associate EVs rapidly entry into the recipient cells mainly through macropinocytosis pathway and cooperated with caveolin- and clathrin-mediated endocytosis. Importantly, EVs-mediated increased SLAM expression enhance PPRVreplication levelsas well as the expression of various cytokines related to SLAM signaling pathway in the recipient cells.

Despite recent advances in our understanding of the interplay between EVs and virus, much of this information has been obtained from impure EVs preparations, which have confounded interpretation of findings. For example, it is well known that some viral particles have size, buoyant densities, or sedimentation velocities similar to those of EVs, it is difficult to separate the two populations completely. CD63 or composite magnetic bead purification is by far the best method to completely separate EVs and virions [26,55]. A combination of density gradient centrifugation and CD63 immunomagnetic bead affinity has recently been described [26]. In this study, EVsin the supernatants of PPRV infected cells were isolated by density gradient centrifugation technique combined with CD63 immunomagnetic bead affinity. Electron microscopy, Western blot and NTA analysis confirmed that the purified EVs were not contaminated with free PPRV virions.It is should be noted that, although EVs used in this study were isolated byCD63 immunoaffinity capture, we do not exclude the possible role of other EVs, for example, prepared by CD81 immunoaffinity capture, in PPRV infection.

Many viral infected cells secrete EVs that differ in content from those secreted from normal cells, and that the changed compositions of EVs derived from viral infected cell confer novel functionalities, such as facilitating viral spread and viral evasion of host cell defenses [32,54,56–58]. Our LC-MS/MS analysis showed that 151 caprine proteins differentially expressed in the purified EVs derived from PPRV-infected goat PBMCscompared to that from Mock-infected cells. GO and COG/KOG analysis showed that these host proteins were associated with translation, posttranslational modification, intracellular trafficking, and cytoskeleton. Further study is needed to determine the role of these differentially expressed proteinscontained in EVs in facilitating viral pathogenesis or host immune responses. Moreover, given the findings in this study that the high levels of PPRV H protein carried in PPRV associate EVs and the critical role of PPRV H protein in stimulating SLAM expression and innate immune response [16,59], we focused on the role of PPRV associate EVs in regulating SLAM expression in the recipient cells.

It has previously been demonstrated that PPRV infection induced transient increased SLAM expression [16,60,61]. However, the expression of SLAM in ruminant in responses to PPRV infection is not yet fully understood. Here, we demonstrate that the EVs isolated from PPRV-infected cells can enhance SLAM expression in the recipient cells. These findings are consistent with a suggestion that PPRV-infected cells may contribute to the regulation of SLAM receptor expression on adjacent cells via intercellular communication [16].

To date, information on EVs secretion levels induced by virus infection remains limited [62,63]. Our data reveal that PPRV infection upregulated the secretion levels of EVs and enhanced EVs-mediated SLAM expression in the recipient cells, while blocking EVs release by GW4869 impairs EVs-mediated SLAM expression in a GW4869dose-dependent manner. Although the precise mechanism underlying the increased EVs release in response to PPRV infection is not known, the upregulatedEVs release levels may contribute to enhancing SLAM expression in the recipient cells. Furthermore, the critical roles of PPRV H and V protein in the regulation of the innate immune responses have been previously demonstrated [5,59,64–66]. Here, greater secretion levels of PPRVassociatedEVs was detected and PPRV H and V protein were identifiedinthese EVs. Further study is needed to determine the effect of PPRV associate EVs on other cell types responses that cannot be infected with PPRV.

PPRV H protein is responsible for regulating viral adsorption and entry, determining pathogenicity, and generating protective antibodies during PPRV infection [7,59,64,67,68]. Our recently study has revealed that PPRV H can stimulates SLAM expression in goat PBMCs via suppressing miR-218 expression, a novel negative miRNA directly targeting SLAM gene [16]. Here, the fact that PPRV H-containing EVs produced by H-transfected cells and PPRV-infected cells had similar effects on SLAM expression in the recipient cells, which is a strong indication that the cause of these effects is in fact PPRV H protein, as opposed to other EVs constituents. Importantly, an inverse correlation between the expression of miR-218 and SLAM was observed in the recipient cells. In addition, the increased SLAM expression could be impaired by miR-218 mimics, which confirmed that H protein contained in EVs is sufficient to enhance SLAM expression in the recipient cells through suppressing miR-218 expression.

It has been implicated that transient increased SLAM expression during early PPRV infection may associated with virus replication and PPRV induced immunosuppression [8,15–17]. Here, our data clearly showed that PPRV associateEVs enhance PPRV replication by regulating SLAM receptor expression in the recipient cells. Moreover, SLAM signaling has been reported to function as a modifier in immunodeficiency disease [15,18,19]. SLAM is a self-ligand receptor expressed on the activated lymphocytes, macrophages, and dendritic cells. Previous in vitro experiments suggest that SLAM/SLAM interactions stimulated inflammatory cytokines production and plays an important role in T-helper 1 (Th1) differentiation [69]. Our data showed that PPRV associate EVs and PPRV infection has similar effects on the proinflammatory and anti-inflammatory cytokines expression in the recipient cells. These results suggest that the SLAM signaling regulated by PPRV associate EVs may also contribute to PPRV replication levels and PPRV-induced immunosuppression [66,70].

Given the emerging roles of PPRV associate EVs in the enhancement of SLAM receptor expression, it is important to understand the molecular mechanisms by which PPRV associate EVs are internalized into the recipient cells. Although accumulating evidence has shown that endocytosis followed by fusion is the dominant mode for the transfer of EVs to target cells, a detailed mechanism by which EVs are taken up has remained controversial [24,30,45–50]. EVs internalization seems more complicated in nonphagocytes, in which clathrin-mediated and caveolin-mediated endocytosis, macropinocytosis, and some nonclassic lipid raft-dependent endocytosis are involved [32,49,52,71]. It is believed that various combinations of endocytic mechanisms are responsible for EVs entry in different cell types [24,49]. In this study, we found that macropinocytosis serve as mainly route for PPRV associate EVs internalization and cooperated with caveolin- and clathrin-mediated endocytosis to ensure EVs-mediated modulation of SLAM expression. It has been shown that EVs of various cellular origins preferentially target specific cell types [49,72,73]. Further investigation will be required to determine whether PPRV associateEVs are internalized into other caprine cell types via similar routes.

**Fig 8:**
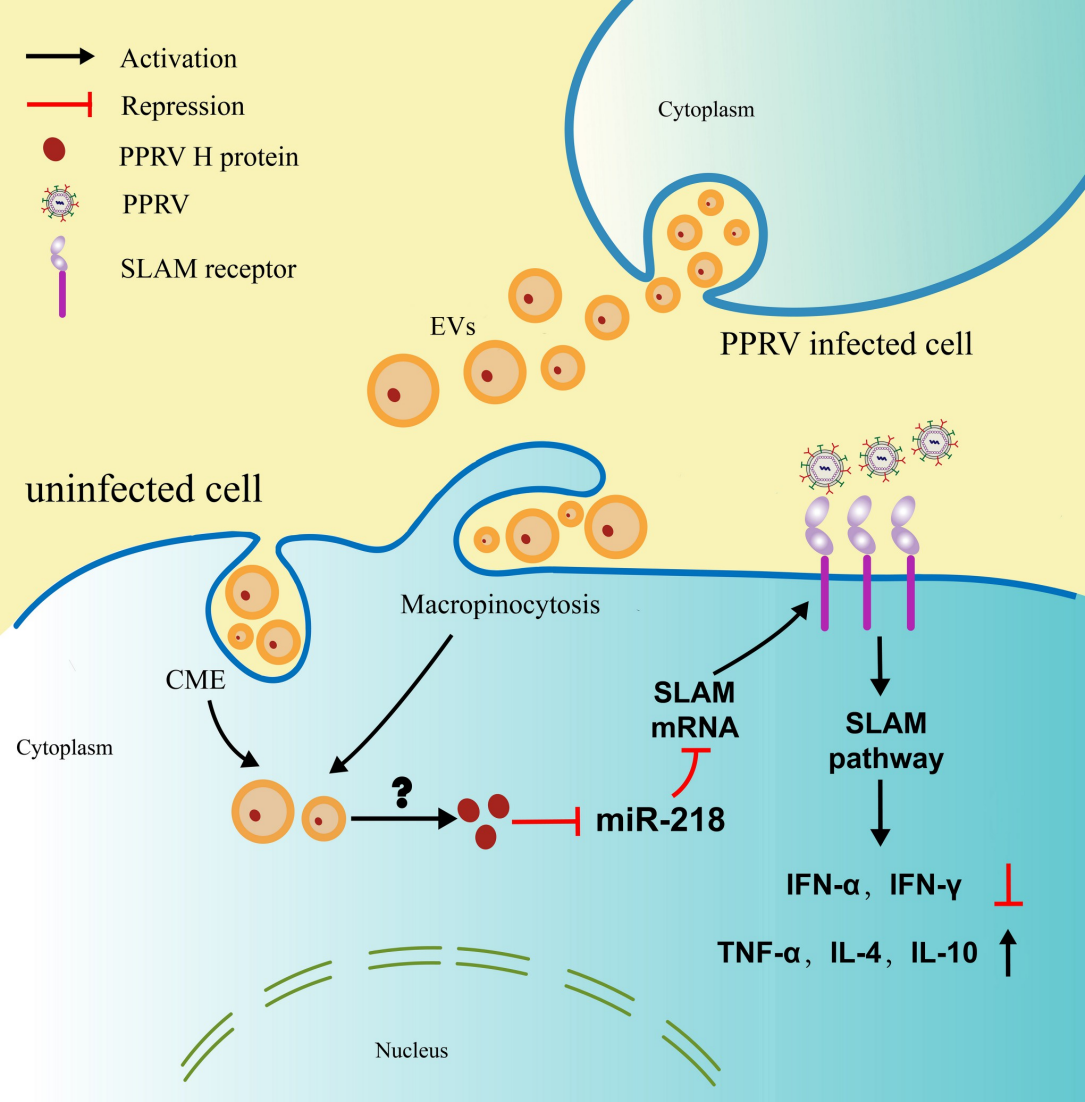
Proposed model for the PPRV H protein-containing extracellular vesicles mediated enhancement of SLAM expression in the recipient cells and regulation of virus invasion. PPRV infection stimulates EVs secretion and EVs entry into the recipient cells mainly via macropinocytosis route and cooperated with caveolin- and clathrin-mediated endocytosis. PPRV H protein contained in extracellular vesicles can enhance miR-218-mediated SLAM expression in the recipient cells, and promote PPRV infection. PPRV associate EVs can regulate SLAM mediated cytokines expression in the recipient cells.

Our model, in which PPRV associated EVs enhance SLAM expression in the recipient cells through suppressing miR-218 expression and facilitate PPRV infection, is shown in Fig 8. In this model, the EVs derived from PPRV infected goat PBMCs enters into the naive recipient cells mainly through macropinocytosis pathway and cooperated with caveolin- and clathrin-mediated endocytosis. PPRV H protein carried in EVs is sufficient to induce SLAM receptor expression in the recipient cells via suppressing miR-218, although how H proteincontained in EVs are released after internalization is not yet fully understand. Importantly, our data reveal that EVs-mediated increased SLAM expression enhance PPRV infectivity as well as the expression of various cytokines related to SLAM signaling pathway in the recipient cells (Fig 8). In summary, we present a strategy used by PPRV to enhance virus replication and escape innate immunity by engaging EVs pathway, which may help us to further understand PPRV pathogenesis.It must, however, be recognized that, althoughthe critical role of EVs in PPRV infection is characterized in this study, cell culture studies are unlikely to reflect interactions that occur between EVs and the recipient cells in vivo environment due to unclear dynamics of EV release and uptake, biodistribution, and half-life [74].A clinical study relating clinical endpoints to biological activity of EVs isolated from plasma of PPRV-infected goats is required to convincingly address this question.

## Materials and methods

### Ethics statement

The animal experiments were carried out in strict accordance with guidelines established by Ethics of Animal Experiments of Northwest A&F University, Yangling, China. All the protocols were approved by this committee (Permit Number: 2014BAD23B11). Healthy 6 months old goats used for blood collection were housed in appropriate containment facilities and had *ad libitum* access to feed and water. Goats were screened for PPRV antibodies using competitive enzyme-linked immunosorbent assay (ELISA) and serum neutralization test and all tested negative.

### PBMCs isolation and virus infection

Goat PBMCs were isolated using Histopaque-1077 (Sigma, USA) by density gradient centrifugation following the manufacturer’s instructions. Then, isolated cells from each goat were suspended into 70 ml RPIM-1640 medium (Hyclone, Logan, UT, USA) supplemented with 10% fetal calf serum (FCS), 100 mg/ml penicillin, and 100 IU/ml streptomycin as described previously [16]. The PPRV vaccine strain, Nigeria 75/1, was obtained from the Lanzhou Veterinary Research Institute, Chinese Academy of Agricultural Sciences (Lanzhou, China). Virus stock was prepared by collecting the infected Vero cell supernatant when cytopathic effect (CPE) affected about 80% of the cells. The virus was harvested by three cycles of freezing and thawing and stored at -80°C and purified by banding on sucrose gradient. The purified virus titers were estimating 50% tissue culture infective doses (TCID_50_) using Vero cells in 96 well micro titer plate.

For virus infection, goat PBMCs were seeded into six well plates at a density of 1×10^5^ cells/ml and were inoculated with Nigeria 75/1 at a multiplicity of infection (MOI) of 1.0. After 1h of adsorption, infected cells were maintained in RPMI-1640 medium (Hyclone, Logan, UT, USA) supplemented with 2% FCS. PBMCs inoculated with similarly purified preparation from triple freeze-thawed Vero cells were used as the mock-infected group. Viral infection in PBMCs was confirmed with CPE were observed under a light microscope at 0, 24, 48, and 72 hpi. Western blot was performed using a polyclonal antibody against PPRV N protein to determine virus replication at the different time points after infection. Three replicates of PPRV- and mock-inoculated cultures were prepared at indicated time point.

### Antibodies and reagents

Anti-PPRV-N, anti-PPRV-H and anti-PPRV-V monoclonal antibody were provided by the China Animal Health and Epidemiology Center (Qingdao, China). The following primary antibodies were used: anti-CD63 (1:1500; Santa Cruz), anti-CD81 (1:1000; Santa Cruz), anti-SLAM (1:2000; Santa Cruz), anti-GAPDH (1:2000; Invitrogen), anti-IL10 (1:1500; ABclonal), anti-IFNa (1:1500; ABclonal), anti-IFNγ (1:1500; Abcam), or anti-TNFa (1:1000; Abcam). Secondary antibodies: HRP-conjugated mouse anti-rabbit IgG (1:15000; Cell Signaling Technology), HRP-conjugated goat anti-mouse IgG (1:20000; Sigma-Aldrich), HRP-conjugated goat anti-rabbit IgG (1:15000; Sigma-Aldrich), PE-conjugated goat anti-rabbit IgG (1:20000; TransGen Biotech), fluorescein isothiocyanate (FITC)-conjugated goat anti-rabbit IgG (1:15000; Sigma-Aldrich), tetramethyl rhodamine isothiocyanate (TRITC)-conjugated goat anti-mouse IgG (1:15000; Sigma-Aldrich).

### Extracellular vesicles isolation and purification

Extracellular vesicles from cell culture supernatants were isolated and characterized as described previously [35]. Briefly, virus-infected cells were washed twice with PBS and then supplemented with RPMI-1640 containing 2% exosome-depleted serum (System Biosciences, USA) and incubated for 2 days. Culture supernatants were collected and processed for EVs isolation by density gradient method [35]. To prepare the discontinuous iodixanol gradient, 40% (w/v), 20% (w/v), 10% (w/v) and 5% (w/v) solutions of iodixanol were made by diluting a stock solution with 0.25 M sucrose/10 mMTris, pH 7.5. The gradient was formed by adding 3 ml of 40% iodixanol solution to a 14 by 89-mm polyallomer tube (Beckman Coulter), followed by careful layering of 3 ml each of 20% and 10% solutions, and 2.5 ml of 5% solution. CCM (500 μl, 1.5 mg protein) was overlaid onto the top of the gradient, and centrifugation performed at 100,000×g for 18 h at 4°C. Twelve individual 1 ml gradient fractions were collected manually (with increasing density). Fractions were diluted with 2 ml PBS and centrifuged at 100,000×g for 3 h at 4°C followed by washing 1 ml PBS, and resuspended in 50 μl PBS. Fractions were monitored for the expression of EVs markers CD63, CD81, TSG101 (System Biosciences, USA) and negative marker Calnexin (San Diego, CA, USA) by Western blot. To determine the density of each fraction, a control gradient containing 500 μl of 0.25 M sucrose/10 mMTris, pH 7.5 was run in parallel. Fractions were collected as described, serially diluted 1:10, 000 with water, and the iodixanol concentration determined by absorbance at 244 nm using a molar extinction coefficient of 320 lg^-1^ cm^-1^. To purify the EVs recovered, CD63-labeled Dynabeads (Invitrogen) were used according to the manufacturer’s instructions[35].Then, EVs were eluted from the Dynabeads using ice-cold 100 nM glycine-HCL (pH 3.0) and immediately neutralized to pH 7.4 with neutralizing buffer (1 M Tris-HCL, pH 8.5). The resulting EVs pellet was subjected to size and concentration measurement by NanoSight NS300 (Malvern Instruments, Westborough, MA) at XiaopengInc (Guangzhou, China).

### Extracellular vesicles quantification assay

Extracellular vesicles concentration was assessed using the EXOCET assay (System Biosciences, Mountain View, CA, USA), according to the manufacturer’s instructions. This is an enzymatic colorimetric assay measuring the absorbance at 405 nm of esterase activity known to be within EVs. The assay was calibrated using a standard EVs preparation (System Biosciences).

### Electron microscopy

The purified EVs were spotted onto Formvar-coated copper grids (200 meshes). The absorbed EVs were fixed in 2% (vol/vol) paraformaldehyde for 5 min at room temperature. After fixation, the grids were directly stained with uranyl acetate for contrast enhancement and then examined using a transmission electron microscope (Hitachi H-7000FA, Lapan).

### Liquid chromatography-tandem mass spectrometry (LC-MS/MS) analysis

For mass spectrometry analysis, the purified EVs were resuspended in 25 μl elution buffer (50 mM glycine, pH 2.8). Proteins were digested with the filter-aided sample preparation (FASP) procedure, as described previously [75]. Briefly, the protein pellet (about 30 μg) was solubilized in 30 μl SDT buffer (4% [mass/vol] SDS, 100 mM Tris-HCl, 1 mM dithiothreitol [DTT] [pH 7.6]) at 90°C for 5 min. The detergent, DTT, and other low-molecular-weight components were removed using 200μl UA buffer (8 M urea, 150 mM Tris-HCl, pH 8.0) with repeated ultrafiltration (Microcon-30kDa centrifugal filter unit). Iodoacetamide (0.05 M, 100μl) in UA buffer was then added to block the reduced cysteine residues, and the samples were incubated for 20 min in the dark. The filter was washed three times with 100 μl of UA buffer and then twice with 100μl of 25 mM NH_4_HCO_3_. Finally, the protein suspension was digested with 2 μg of trypsin (Promega) in 40 μl of 25 mM NH_4_HCO_3_ overnight at 37°C. The resulting peptides were collected as the filtrate. Experiments were performed on a Q Exactive mass spectrometer coupled to an Easy-nLC liquid chromatograph (Proxeon Biosystems).

### Fluorescent labeling of extracellular vesicles

Extracellular vesicles were fluorescently labeled according to the manufacturer’s instructions (Life Technologies, Carlsbad, CA). Briefly, 1 ml of fractionated EVs (100 ng/ml) was incubated with 6μl of a 100 μM stock solution of PKH26 (Life Technologies, Carlsbad, CA) for 1h in the dark at room temperature with gentle agitation. Then, the EVs were isolated again according to the EVs extraction method to remove the excess dye.

### Flow cytometric assay

For EVs flow cytometry analysis, EVs freshly isolated from cell culture supernatants were labeled with a commercially available CD63 and CD81 detection kit (Invitrogen) according to the manufacturer’s instructions. Briefly, 50μl of the pre-enriched EVs were mixed with dynabeads coated with anti-CD63 or CD81 antibody. The mixture was incubated overnight under gentle agitation at 4°C. After several washing steps, EVs bound to antibody beads were resuspended in 300 μl PBS with 0.1% BSA. Then, 100μl of bead-bound EVs were incubated with 4μl anti-CD63-FITC and anti-CD81-PE or matching isotype control (BioLegends). After 45 min, the labeled EVs were washed twice and resuspended in 500μl PBS with 0.1% BSA. Then, EVs were analyzed on a FACSCalibur (BD Biosciences, San Jose, CA). The SLAM expression on cells were also analyzed by flow cytometry as described previously [16].

### Nanoparticle tracking analysis

Isolated EVs were examined with the Nanosight N3000 system for numbers and size distribution and the data were analyzed by NTA 3.2 Dev Build 3, 2, 16 [26].

### Confocal immunofluorescence microscopy

Following the indicated treatments, goat PBMCs were washed four times with PBS and fixed in 4% paraformaldehyde. The cells washed again four times with PBS and treated with 0.1% Triton X-100 for 15 min. The cells were then incubated with 1% bovine serum albumin (BSA; Sigma-Aldrich, A7906) and the appropriate primary antibodies for 1h at 37°C. Then, the cells were washed and incubated simultaneously with FITC- or Cy3-cojugated secondary antibodies. Finally, the cells were treated with a Hoechst 33342 (Sigma-Aldrich, B2261) solution for 5 min and analyzed under a confocal microscope (CLSM; Leica SP8, Germany) as described previously [16].

### Real-time quantitative PCR analysis

Total RNA was extracted from goat PBMCs using Trizol reagent (Invitrogen, Waltham, MA, USA) according to the manufacturer’s instructions. RNA was then reversed using Superscript III (Invitrogen) and random primers (Invitrogen). Real-time quantitative PCR was carried out using a ABI 7500 System (Applied Biosystems, Warrington, UK) and Power SYBR Green PCR Master Mix (Applied Biosystems). The sequences of the primers and reaction conditions for SLAM and GAPDH genes, as well as miR-218 expression detection have been described previously [16]. Goat cytokines gene mRNA expression were also detected and the sequences of the primers and reaction conditions were used as previously described [76].

### Western blot analysis

Protein homogenates from the cells were extracted as previously described [5]. Briefly, the cells were lysed for 20 min on ice-cold lysis buffer (Roche). The lysates were centrifuged at 12,000 ×g for 20 min at 4°C to obtain a clear lysate. The protein content of each sample was determined using the BCA Protein Assay Kit (Thermo Scientific). Then, equal amounts of protein were separated on a 12% SDS-polyacrylamide gel and transferred to polyvinylidene difluoride membranes. Membranes were probed overnight at 4°C with primary antibodies. The bands were visualized using horseradish peroxidase (HRP)-conjugated goat anti-mouse IgG (1:15000, Boster) or goat anti-rabbit IgG (1:20000, Boster) prior to the ECL protocol (Amersham Biosciences, Piscataway, NJ, USA). As an internal standard, all membranes stripped with primary antibodies were reprobed with anti-GAPDH antibody (Invitrogen). Changes in protein expression were determined after normalizing the band intensity of each lane to that of GAPDH. Signal was visualized using Konica SRX 101A developer (Konica Minolta Medical Imaging, Wayne, NJ, USA) and the Quantity One software (Bio-Rad, Mississauga, ON, Canada) was used for densitometrical analysis.

### Transient transfection of miRNA

Goat PBMCs were grown to logarithmic phase in six well plates with antibiotic-free medium the day before transfection. The miRNA transfection, including miR-218 mimic and mimic control (MC), was performed with LipofectamineRNAiMAX (Life Technologies, USA) on cells of 50% confluence according to the manufacturer’s protocol. The final concentrations of miR-218 mimic, or MC (RiboBio, Guangzhou. China) was 100 nM. The effect of transfection was examined by quantitative RT-PCR and Western blot.

### Plasmid construct and virus protein expression

PPRV H gene was amplified from PPRV genomic cDNA and cloned into pcDNA3.1 (+) (Invitrogen, V790-20) as described previously [5]. Goat PBMCs were transfected with pcDNA3.1-H plasmid for 48h and harvested and lysed, cell lysates from transfected and untransfected control cells were subjected to Western blot with antibody against PPRV H for expression analysis. The constructed plasmid was sequenced and the correct insertion of gene was verified. The empty vector pcDNA3.1 was used as mock control.

### RNA interference

Small interfering RNAs (siRNAs) targeting SLAM (target site: TGGATAATGCTGGTCCAGT) and scrambled sequences (control siRNA for SLAM: CTTAGGTTACGAATCGTAG) were designed and synthesized by RiboBio Inc. (GuangZhou. China). Small interfering RNAs were then used for silencing the target genes as described previously [16]. Briefly, goat PBMCs were transfected with 50nM siRNA targeting SLAM, or scramble control RNAs by using Lipofectamine 2000 according to manufacturer’s guidelines (Invitrogen, Carlsbad, CA, USA). 48 h after transfection, cells were cultured in RPMI-1640 medium supplemented with 10% FCS for 48 h, and transfected with EVs followed by infected with PPRV at an MOI of 1 for 24 h before the cells were harvested.

### Virus titration

Virus progeny production was determined by titration as described previously [77]. The viral supernatants from goat PBMCs were collected at the indicated time points after virus inoculation, and the TCID_50_ was calculated by the Reed-Muench method.

### Statistical analysis

All values are expressed as the arithmetic means of triplicates ± standard deviation (SD) from three independent experiments. Significance was determined by one-way ANOVA with a Dunnett-posttests, or by the Student paired *t* test. Values of *P*<0.05 were considered statistically significant.

## Supporting information

S1 Table Details of differentially expressed proteins in PPRV associated extracellular vesicles (EVs) versus mock associated EVs. The content of EVs isolated from PPRV-infected cells and from Mock-infected cells was subjected to liquid chromatography-tandem mass spectrometry (LC-MS/MS) analysis.
(DOCX)
