# Supplementary material for: Extracellular vesicles derived from PPRV-infected cells enhance signaling lymphocyte activation molecular (SLAM) receptor expression and facilitate virus infection
Source: PLoS Pathog. 2022 Sep 9;18(9):e1010759. doi: 10.1371/journal.ppat.1010759 (PMC9491601; doi:10.1371/journal.ppat.1010759)
Supplement: S1 Table — The content of EVs isolated from PPRV-infected cells and from Mock-infected cells was subjected to liquid chromatography-tandem mass spectrometry (LC-MS/MS) analysis. (DOCX) [file ppat.1010759.s001.docx]

**Supplementary file**

**Table1 Details of differentially expressed proteins in PPRV associated extracellular vesicles (EVs) versus mock associated EVs**

| **No.** | **protein name** | **Up down**  **regulation** | **Log2 ratio** | **P value** |
| --- | --- | --- | --- | --- |
| 1 | protein disulfide-isomerase A3 precursor [Capra hircus] | Up | 1.051 | 0 |
| 2 | T-complex protein 1 subunit theta isoform X2 [Capra hircus] | Up | 1.439 | 0 |
| 3 | tubulin alpha-1D chain [Capra hircus] | Up | 1.929 | 0 |
| 4 | coatomer subunit alpha isoform X2 [Capra hircus] | Up | 1.543 | 0 |
| 5 | T-complex protein 1 subunit gamma [Capra hircus] | Up | 1.735 | 0 |
| 6 | serine--tRNA ligase, cytoplasmic [Capra hircus] | Up | 2.686 | 0 |
| 7 | peroxiredoxin-1 [Capra hircus] | Up | 3.320 | 0 |
| 8 | 40S ribosomal protein S8 [Capra hircus] | Up | 1.675 | 0 |
| 9 | proteasome subunit beta type-2 [Capra hircus] | Up | 1.446 | 0 |
| 10 | septin-7 isoform X5 [Capra hircus] | Up | 2.005 | 0 |
| 11 | staphylococcal nuclease domain-containing protein 1 [Capra hircus] | Up | 2.247 | 0 |
| 12 | myotrophin [Capra hircus] | Up | 1.764 | 0 |
| 13 | T-complex protein 1 subunit beta [Capra hircus] | Up | 2.097 | 0 |
| 14 | cullin-associated NEDD8-dissociated protein 1 [Capra hircus] | Up | 4.117 | 0 |
| 15 | ATP synthase subunit beta, mitochondrial [Capra hircus] | Up | 6.954 | 0 |
| 16 | proliferation-associated protein 2G4 isoform X2 [Capra hircus] | Up | 1.760 | 0 |
| 17 | 60S ribosomal protein L3 [Capra hircus] | Up | 1.768 | 0 |
| 18 | elongation factor 2 [Capra hircus] | Up | 3.475 | 0 |
| 19 | 26S proteasome non-ATPase regulatory subunit 5 [Capra hircus] | Up | 1.987 | 0 |
| 20 | elongation factor 1-alpha 1 [Capra hircus] | Up | 3.123 | 0 |
| 21 | T-complex protein 1 subunit alpha [Capra hircus] | Up | 1.554 | 0 |
| 22 | 60S ribosomal protein L4 [Capra hircus] | Up | 1.222 | 0 |
| 23 | pyruvate kinase PKM isoform X1 [Capra hircus] | Up | 3.504 | 8.31E-304 |
| 24 | proteasome subunit beta type-5 [Capra hircus] | Up | 1.946 | 1.86E-290 |
| 25 | peptidyl-prolyl cis-trans isomerase B [Capra hircus] | Up | 1.331 | 4.92E-257 |
| 26 | T-complex protein 1 subunit eta [Capra hircus] | Up | 2.139 | 1.32E-145 |
| 27 | ubiquitin-40S ribosomal protein S27a [Capra hircus] | Up | 1.247 | 5.08E-120 |
| 28 | malate dehydrogenase, cytoplasmic [Capra hircus] | Up | 1.851 | 1.12E-98 |
| 29 | poly(rC)-binding protein 1 [Capra hircus] | Up | 2.097 | 9.35E-89 |
| 30 | 78 kDa glucose-regulated protein isoform X1 [Capra hircus] | Up | 2.463 | 6.43E-85 |
| 31 | heat shock cognate 71 kDa protein isoform X1 [Capra hircus] | Up | 1.486 | 4.18E-82 |
| 32 | 60S ribosomal protein L27a [Capra hircus] | Up | 1.784 | 1.02E-81 |
| 33 | 40S ribosomal protein S3 [Capra hircus] | Up | 4.279 | 1.32E-71 |
| 34 | actin-related protein 2/3 complex subunit 3 [Capra hircus] | Up | 1.486 | 2.29E-68 |
| 35 | vacuolar protein sorting-associated protein 35 [Capra hircus] | Up | 1.727 | 4.54E-65 |
| 36 | 26S proteasome non-ATPase regulatory subunit 7 [Capra hircus] | Up | 2.392 | 1.04E-61 |
| 37 | calpain small subunit 1 [Capra hircus] | Up | 1.951 | 8.22E-61 |
| 38 | 60S ribosomal protein L18 [Capra hircus] | Up | 2.009 | 9.74E-61 |
| 39 | eukaryotic initiation factor 4A-I [Capra hircus] | Up | 3.645 | 7.24E-57 |
| 40 | ATP-citrate synthase isoform X3 [Capra hircus] | Up | 3.024 | 2.73E-56 |
| 41 | ras-related protein Rab-5C [Capra hircus] | Up | 2.078 | 8.67E-56 |
| 42 | protein disulfide-isomerase [Capra hircus] | Up | 2.032 | 2.41E-54 |
| 43 | T-complex protein 1 subunit epsilon [Capra hircus] | Up | 1.288 | 5.55E-54 |
| 44 | 60S ribosomal protein L10a [Capra hircus] | Up | 3.410 | 9.16E-54 |
| 45 | heat shock protein HSP 90-beta [Capra hircus] | Up | 2.895 | 4.36E-53 |
| 46 | chloride intracellular channel protein 1 [Capra hircus] | Up | 1.636 | 1.12E-50 |
| 47 | major vault protein [Capra hircus] | Up | 5.040 | 7.65E-50 |
| 48 | phosphoglycerate mutase 1 [Capra hircus] | Up | 1.907 | 5.49E-47 |
| 49 | elongation factor 1-gamma [Capra hircus] | Up | 2.173 | 2.97E-43 |
| 50 | phosphoglycerate kinase 1 [Capra hircus] | Up | 1.384 | 3.97E-43 |
| 51 | 60S acidic ribosomal protein P0 isoform X1 [Capra hircus] | Up | 2.577 | 4.49E-40 |
| 52 | 60S ribosomal protein L8 [Capra hircus] | Up | 1.226 | 1.92E-39 |
| 53 | 26S proteasome non-ATPase regulatory subunit 3 [Capra hircus] | Up | 3.995 | 1.97E-39 |
| 54 | lanC-like protein 1 [Capra hircus] | Up | 1.226 | 2.74E-38 |
| 55 | tubulin beta chain [Capra hircus] | Up | 2.173 | 3.86E-38 |
| 56 | heat shock 70 kDa protein 1B [Capra hircus] | Up | 1.814 | 1.45E-37 |
| 57 | asparagine--tRNA ligase, cytoplasmic isoform X2 [Capra hircus] | Up | 3.115 | 1.76E-36 |
| 58 | ATP synthase subunit alpha, mitochondrial [Capra hircus] | Up | 4.174 | 8.05E-36 |
| 59 | septin-2 [Capra hircus] | Up | 1.327 | 9.73E-34 |
| 60 | hemoglobin subunit alpha-1 [Capra hircus] | Up | 1.517 | 4.43E-33 |
| 61 | T-complex protein 1 subunit zeta [Capra hircus] | Up | 1.776 | 6.00E-33 |
| 62 | heat shock protein beta-1 [Capra hircus] | Up | 1.366 | 5.09E-30 |
| 63 | serine/threonine-protein phosphatase 2B catalytic subunit beta isoform isoform X8 [Capra hircus] | Up | 1.292 | 9.27E-30 |
| 64 | 60S acidic ribosomal protein P2 [Capra hircus] | Up | 1.977 | 2.66E-29 |
| 65 | coronin-1B [Capra hircus] | Up | 1.094 | 5.92E-29 |
| 66 | serine/threonine-protein phosphatase PP1-alpha catalytic subunit [Capra hircus] | Up | 1.735 | 1.50E-28 |
| 67 | ubiquitin-like modifier-activating enzyme 1 [Capra hircus] | Up | 1.111 | 3.81E-28 |
| 68 | glucose-6-phosphate 1-dehydrogenase isoform X4 [Capra hircus] | Up | 4.407 | 5.20E-28 |
| 69 | mitogen-activated protein kinase 1 isoform X1 [Capra hircus] | Up | 3.835 | 9.73E-34 |
| 70 | uncharacterized protein LOC102169433 [Capra hircus] | Up | 1.476 | 4.43E-33 |
| 71 | ATP-dependent 6-phosphofructokinase, liver type [Capra hircus] | Up | 1.104 | 6.00E-33 |
| 72 | 60S ribosomal protein L5 isoform X2 [Capra hircus] | Up | 2.717 | 5.09E-30 |
| 73 | 4-trimethylaminobutyraldehyde dehydrogenase [Capra hircus] | Up | 1.420 | 9.27E-30 |
| 74 | 26S proteasome non-ATPase regulatory subunit 2 [Capra hircus] | Up | 2.762 | 2.66E-29 |
| 75 | cAMP-dependent protein kinase type II-beta regulatory subunit [Capra hircus] | Up | 2.436 | 5.92E-29 |
| 76 | endoplasmin [Capra hircus] | Up | 2.847 | 1.50E-28 |
| 77 | cytosol aminopeptidase [Capra hircus] | Up | 1.348 | 3.81E-28 |
| 78 | general vesicular transport factor p115 isoform X4 [Capra hircus] | Up | 1.457 | 5.20E-28 |
| 79 | septin-11 isoform X6 [Capra hircus] | Up | 1.164 | 9.73E-34 |
| 80 | heterogeneous nuclear ribonucleoprotein D0 isoform X4 [Capra hircus] | Up | 1.612 | 4.43E-33 |
| 81 | receptor of activated protein C kinase 1 [Capra hircus] | Up | 3.754 | 6.00E-33 |
| 82 | dynamin-2 isoform X7 [Capra hircus] | Up | 1.362 | 5.09E-30 |
| 83 | ras-related protein Rab-14 isoform X1 [Capra hircus] | Up | 1.134 | 9.27E-30 |
| 84 | transitional endoplasmic reticulum ATPase [Capra hircus] | Up | 1.723 | 2.66E-29 |
| 85 | transmembrane emp24 domain-containing protein 10 [Capra hircus] | Up | 2.392 | 5.92E-29 |
| 86 | annexin A2 [Capra hircus] | Up | 5.256 | 1.50E-28 |
| 87 | dynactin subunit 1 isoform X10 [Capra hircus] | Up | 2.491 | 3.81E-28 |
| 88 | exportin-1 isoform X1 [Capra hircus] | Up | 4.604 | 5.20E-28 |
| 89 | T-complex protein 1 subunit delta [Capra hircus] | Up | 1.620 | 9.73E-28 |
| 90 | actin-related protein 2 [Capra hircus] | Up | 1.381 | 8.62E-27 |
| 91 | complement C1q subcomponent subunit C [Capra hircus] | Up | 1.278 | 2.21E-26 |
| 92 | annexin A4 [Capra hircus] | Up | 2.313 | 2.87E-26 |
| 93 | 14-3-3 protein theta [Capra hircus] | Up | 2.178 | 3.02E-26 |
| 94 | chloride intracellular channel protein 4 [Capra hircus] | Up | 2.408 | 5.70E-26 |
| 95 | tubulin beta-4B chain [Capra hircus] | Up | 1.942 | 5.14E-25 |
| 96 | ATP-dependent 6-phosphofructokinase, platelet type [Capra hircus] | Up | 1.264 | 2.19E-24 |
| 97 | calpain-2 catalytic subunit [Capra hircus] | Up | 1.181 | 2.50E-24 |
| 98 | alpha-enolase isoform X1 [Capra hircus] | Up | 1.316 | 6.48E-24 |
| 99 | peroxiredoxin-6 [Capra hircus] | Up | 1.131 | 4.14E-23 |
| 100 | serine/threonine-protein phosphatase 2A catalytic subunit beta isoform [Capra hircus] | Up | 2.491 | 1.14E-22 |
| 101 | GTP-binding nuclear protein Ran [Capra hircus] | Up | 2.425 | 2.05E-21 |
| 102 | signal transducer and activator of transcription 1-alpha/beta [Capra hircus] | Up | 1.581 | 5.37E-21 |
| 103 | actin-related protein 3 [Capra hircus] | Up | 1.019 | 6.38E-21 |
| 104 | alpha-actinin-4 isoform X1 [Capra hircus] | Up | 1.691 | 1.38E-20 |
| 105 | 60S ribosomal protein L13a [Capra hircus] | Up | 1.395 | 9.98E-20 |
| 106 | serine/threonine-protein phosphatase 2A 65 kDa regulatory subunit A alpha isoform [Capra hircus] | Up | 2.247 | 3.56E-19 |
| 107 | septin-9 isoform X4 [Capra hircus] | Up | 1.723 | 9.78E-19 |
| 108 | clathrin heavy chain 1 isoform X4 [Capra hircus] | Up | 2.408 | 1.88E-18 |
| 109 | 26S proteasome non-ATPase regulatory subunit 11 [Capra hircus] | Up | 3.882 | 2.39E-18 |
| 110 | platelet-activating factor acetylhydrolase IB subunit alpha [Capra hircus] | Up | 1.600 | 3.26E-17 |
| 111 | nucleoside diphosphate kinase B [Capra hircus] | Up | 1.573 | 5.68E-17 |
| 112 | importin subunit beta-1 [Capra hircus] | Up | 3.194 | 4.04E-17 |
| 113 | vesicle-fusing ATPase isoform X2 [Capra hircus] | Up | 1.760 | 7.12E-16 |
| 114 | ras GTPase-activating-like protein IQGAP1 [Capra hircus] | Up | 3.907 | 2.14E-15 |
| 115 | cytoplasmic dynein 1 heavy chain 1 [Capra hircus] | Up | 1.185 | 1.38E-15 |
| 116 | heat shock protein HSP 90-alpha [Capra hircus] | Up | 1.843 | 2.70E-15 |
| 117 | ras-related protein Rab-7a isoform X1 [Capra hircus] | Up | 1.002 | 3.13E-15 |
| 118 | 60S ribosomal protein L14 [Capra hircus] | Up | 2.355 | 2.80E-14 |
| 119 | apolipoprotein D [Capra hircus] | Down | -1.573 | 4.01E-197 |
| 120 | complement C1q subcomponent subunit B [Capra hircus] | Down | -2.450 | 1.22E-177 |
| 121 | collagen alpha-2(I) chain [Capra hircus] | Down | -1.569 | 3.04E-164 |
| 122 | collectin-11 isoform X2 [Capra hircus] | Down | -1.905 | 1.38E-110 |
| 123 | pantetheinase isoform X2 [Capra hircus] | Down | -1.012 | 7.94E-98 |
| 124 | hemopexin [Capra hircus] | Down | -1.333 | 8.56E-90 |
| 125 | plasma serine protease inhibitor [Capra hircus] | Down | -1.388 | 2.45E-62 |
| 126 | protein kinase C beta type isoform X2 [Capra hircus] | Down | -1.056 | 3.35E-60 |
| 127 | coagulation factor XI [Capra hircus] | Down | -1.108 | 1.18E-59 |
| 128 | plastin-2 [Capra hircus] | Down | -1.056 | 4.30E-48 |
| 129 | methylmalonyl-CoA mutase, mitochondrial [Capra hircus] | Down | -1.604 | 8.37E-27 |
| 130 | histone H1.5 [Capra hircus] | Down | -3.158 | 1.20E-26 |
| 131 | platelet glycoprotein IX [Capra hircus] | Down | -1.133 | 3.57E-13 |
| 132 | histone H1.3 [Capra hircus] | Down | -2.708 | 4.12E-13 |
| 133 | histone H2A type 1-H [Capra hircus] | Down | -1.355 | 1.52E-12 |
| 134 | histone H2B type 1-K [Capra hircus] | Down | -1.366 | 2.23E-12 |
| 135 | uncharacterized protein LOC102183743 [Capra hircus] | Down | -1.411 | 2.26E-12 |
| 136 | insulin-like growth factor-binding protein complex acid labile subunit [Capra hircus] | Down | -1.009 | 3.13E-12 |
| 137 | ADP-ribosylation factor 4 [Capra hircus] | Down | -1.276 | 6.18E-12 |
| 138 | lymphocyte function-associated antigen 3 isoform X3 [Capra hircus] | Down | -1.114 | 8.67E-12 |
| 139 | protein kinase C and casein kinase substrate in neurons protein 2 isoform X2 [Capra hircus] | Down | -1.419 | 1.47E-11 |
| 140 | histone H2A.Z [Capra hircus] | Down | -1.544 | 6.07E-11 |
| 141 | core histone macro-H2A.1 isoform X4 [Capra hircus] | Down | -1.133 | 7.33E-11 |
| 142 | complement factor D [Capra hircus] | Down | -1.262 | 9.99E-11 |
| 143 | collagen alpha-2(VI) chain isoform X1 [Capra hircus] | Down | -1.133 | 1.09E-10 |
| 144 | gelsolin isoform X4 [Capra hircus] | Down | -2.786 | 2.79E-10 |
| 145 | CD109 antigen [Capra hircus] | Down | -1.221 | 3.69E-10 |
| 146 | thyroglobulin [Capra hircus] | Down | -1.012 | 1.03E-09 |
| 147 | peptidoglycan recognition protein 1 [Capra hircus] | Down | -2.102 | 1.46E-09 |
| 148 | 40S ribosomal protein S9 [Capra hircus] | Down | -1.671 | 2.12E-09 |
| 149 | cytochrome b reductase 1 [Capra hircus] | Down | -1.003 | 4.08E-09 |
| 150 | integrin alpha-6 isoform X2 [Capra hircus] | Down | -1.400 | 1.15E-08 |
| 151 | integrin alpha-IIb isoform X2 [Capra hircus] | Down | -1.419 | 3.22E-08 |
